# Dimeric allostery mechanism of the plant circadian clock photoreceptor ZEITLUPE

**DOI:** 10.1371/journal.pcbi.1009168

**Published:** 2021-07-26

**Authors:** Francesco Trozzi, Feng Wang, Gennady Verkhivker, Brian D. Zoltowski, Peng Tao

**Affiliations:** 1 Department of Chemistry, Center for Research Computing, Center for Drug Discovery, Design, and Delivery (CD4), Southern Methodist University, Dallas, Texas, United States of America; 2 Graduate Program in Computational and Data Sciences, Schmid College of Science and Technology, Chapman University, Orange, California, United States of America; 3 Chapman University School of Pharmacy, Irvine, California, United States of America; US Army Medical Research and Materiel Command: US Army Medical Research and Development Command, UNITED STATES

## Abstract

In *Arabidopsis thaliana*, the Light-Oxygen-Voltage (LOV) domain containing protein ZEITLUPE (ZTL) integrates light quality, intensity, and duration into regulation of the circadian clock. Recent structural and biochemical studies of ZTL indicate that the protein diverges from other members of the LOV superfamily in its allosteric mechanism, and that the divergent allosteric mechanism hinges upon conservation of two signaling residues G46 and V48 that alter dynamic motions of a Gln residue implicated in signal transduction in all LOV proteins. Here, we delineate the allosteric mechanism of ZTL via an integrated computational approach that employs atomistic simulations of wild type and allosteric variants of ZTL in the functional dark and light states, together with Markov state and supervised machine learning classification models. This approach has unveiled key factors of the ZTL allosteric mechanisms, and identified specific interactions and residues implicated in functional allosteric changes. The final results reveal atomic level insights into allosteric mechanisms of ZTL function that operate via a non-trivial combination of population-shift and dynamics-driven allosteric pathways.

## Author summary

From growth to flowering, many aspects of plants development are regulated by daily and seasonal changes in light intensity and duration. These circadian clocks employ light sensing proteins to adjust their functional states responding to different wavelengths and light intensities that result from daily and seasonal oscillations in our environment. The mechanistic insights into how the absorption of light causes changes in their functional states remain elusive for many circadian clock proteins. ZEITLUPE (ZTL) is a circadian clock protein found in *Arabidopsis thaliana*. Previous experimental studies demonstrated that ZTL functions as a dimer, which could adopt either parallel or anti-parallel orientations depending on the presence of blue-light or darkness, respectively. In this study, the authors investigated the allosteric mechanism of ZTL in both parallel and anti-parallel dimeric states through computational means. Two double mutants with significant functional deviation, G46S:G80R and V48I:G80R, were also studied as comparison. As the results, the authors provided mechanistic and dynamic models with atomistic details underlying the functions of this circadian clock protein.

## Introduction

Light-Oxygen-Voltage (LOV) domains are widespread in nature, where they couple sensing of UVA/Blue light into regulation of diverse modes of biological activity.[1–3] Central to their function is an innate ability to couple cofactor chemistry to dynamic allosteric changes in protein conformation to regulate either protein-protein interactions, or activity of signal-transduction domains N- or C-terminal to the LOV domain.[2] The ability of LOV domains to regulate diverse signaling elements has led to their widespread use in optogenetic tools,[4–6] and has identified them as model proteins for studying allostery, the change of the protein structural conformation or states distribution due to a perturbation of a non-functional secondary site.[7–10]

Structural and computational approaches have been used to study LOV signal transduction in a number of systems.[11–20] These approaches have identified a mechanism of signal transduction dependent on three conserved elements: i) UVA/Blue-light triggers formation of a covalent adduct between a flavin cofactor (Riboflavin/FMN/FAD), and a conserved Cysteine residue resulting in sp^3^ hybridization of the flavin C4a position and protonation of the flavin N5 position[2,15]; ii) N5 protonation causes a conserved Gln residue, coplanar to the isoalloxazine ring (buried conformation), to flip to optimize H-bonding interactions to N5, and alter H-bonding interactions with elements in the A*β*-strand[11,12]; iii) The Gln flip and altered H-bonding interactions induce conformational changes within a central five-stranded *β*-sheet and/or N/C-terminal extensions to the core LOV domain (Ncap/Ccap)[13,16]. The resulting consensus mechanism indicates that the conserved Gln residue is indispensable for LOV-signaling, and N5 protonation is both necessary and sufficient for LOV allostery.[11,21,22]

Recent structural and biochemical studies of the *Arabidopsis thaliana* circadian clock photoreceptor ZEITLUPE (ZTL) have called the consensus LOV signaling mechanism into question, and have suggested that LOV allostery may be more fluid across the LOV superfamily.[16,23] Structures of ZTL proteins in the dark- and light-states indicate divergent signaling elements compared to other LOV proteins: i) The conserved Gln residue (Gln154) adopts a dynamic and heterogeneous population rotating between buried and exposed conformations in the dark. Subsequent photoactivation biases the Gln population towards the buried conformation seen in all other LOV-structures to induce downstream signal transduction[16,23]. ii) Q154L variants are tolerated in ZTL and are naturally present in ZTL family members[23]. iii) Evolutionary analysis revealed that the altered signaling pathway was dependent on conservation of two residues in the A*β*-strand, Gly46 and Val48, that are co-conserved in all ZTL proteins[16]. Further, examination of other LOV structures with Gly residues at the position equivalent to Gly46 revealed altered Gln dynamics in these proteins, thereby highlighting conservation of allosteric motions linking the C-terminal Gln, to N-terminal conformational changes dependent on the residue identity at position 46 in ZTL[23–25].

**Fig 1:**
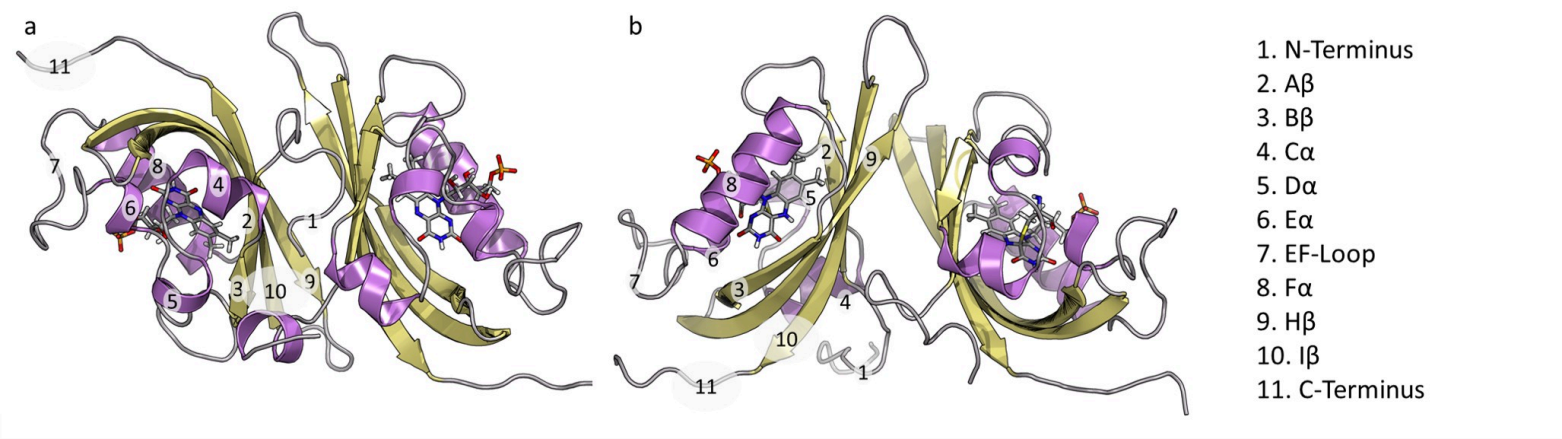
Crystal structures of ZTL dimers: a) Anti-parallel dark state; b) Parallel light state.

Examination of G46S and G46A structures revealed coupling between C-terminal Gln dynamics, the residue identity at position 46, and global conformational changes.[16,23] G46S and G46A structures mimic a light-state like orientation of the active site Gln that is coupled to the movement of a conserved Phe residue (Phe156), C-terminal salt-bridge formation, and the movement of the Ncap. Specifically, rotation of Gln154 towards the buried conformation disrupts contacts to the N/Ccap to induce a 180° rotation about the LOV-dimer interface (Fig 1). However, these observations relied on static structures of allosteric variants that may block observation of dynamic motions necessary for light/dark regulation of ZTL structure, and may not necessarily reflect allostery in WT proteins. Further, the use of allosteric variants to trap specific ZTL configurations only allows for snapshots of starting and end points in an allosteric process, and not the dynamic motions gating the altered mechanisms of signal transduction, thereby limiting understanding of how the ZTL allosteric landscape diverges from other LOV proteins.

To surpass this limitation and obtain a dynamical description of ZTL allostery, exploring the conformations that lie in between the starting and end points in the allosteric process to ultimately extrapolate the structural changes during this process is necessary. In recent years, computational approaches to advance a quantitative characterization of allosteric mechanism in proteins have been developed. Molecular dynamics (MD) simulations are a useful tool to explore the conformational landscape of a protein by providing the evolution of a system over time at an atomistic level.[26–28] Combined with MD simulations, Markov state model (MSM) approaches can provide connectivity maps of states on the free energy landscape, estimate the effect of allosteric perturbations on the conformational equilibrium, and rigorously describe kinetics of allosteric transitions.[19,29–34]

Herein, we employ atomistic simulations that integrate Markov State modeling, and supervised machine learning classification models to elucidate a dynamic allosteric process gating ZTL function. These results confirm a complex and dynamic ZTL landscape that links sequence motifs within the A*β* and I*β* strands to an insertion between the E/F helices (E-F loop), which drives the reorientation of the ZTL LOV-dimer. The results provide keen insight into the role of Gly46 and Val48 mutations in disrupting ZTL allostery that have implications for plant physiology, and broader impacts to LOV allostery.

The ensemble of computational tools employed in this study enabled us to validate and integrate otherwise inaccessible information to the experimental investigations of ZTL allostery. The MD simulations support the stability of dimer complexes, and also reveal the propagation of the allosteric perturbation starting from the flavin photoreceptor to the overall protein structure.

The remainder of the paper is organized as follows. First, it is presented how Gln154 responds to the change in state of the flavin and the impact of G46S and V48I variants on Gln154 dynamics. Then how the different conformations that Gln154 adopts impact the interactions between residues in the functional N- and C-termini are investigated. Several intramolecular interactions that could be crucial to propagate the signal throughout the protein are also investigated. Particularly, how these intramolecular interactions affect the population of functional metastable states on the conformational landscape of the protein is evaluated. Towards these goals we adopted the dimensionality reduction technique tICA and Markov state modeling. Lastly, with the use of Machine Learning we elucidated characteristic structural changes that correlate with different metastable states involved in ZTL allosteric process.

## Results

To elucidate a model of ZTL allostery, we performed a series of MD simulations on ZTL structures for either wild-type (WT) or allosteric variant forms of the isolated LOV domain. Specifically, we simulated the WT dark-state in an anti-parallel conformation (PDB ID: 5SVG), the light and dark anti-parallel conformations of the V48I:G80R variant that disrupts light-driven allostery (PDB ID 5SVV and 5SVW, respectively), WT and G46S:G80R (PDB ID 6WLP) variants in a parallel conformation that mimic the light-state conformation of Q154, and transient structures for both the anti-parallel and parallel conformations. All systems subjected to the simulations in this study are listed in Table 1. Initial focus was placed on resolving several important limitations of allosteric variant structures. Namely, we anticipated to verify two key aspects of ZTL signaling independent of allosteric variants or crystal contact restraints: 1) Verify stability of the ZTL LOV parallel dimer in WT light-state proteins (Protein Stability Analysis in S1 Text); 2) Examine Q154 conformational states and dynamics in WT and allosteric variants of ZTL LOV dimers.

**Table 1:** ZTL systems subjected to simulations.

ZTL state^a^	Dimer structures^b^	Abbreviation^c^
Native Dark WT	Anti-parallel	dark wt anti
Native Light WT^d^	Anti-parallel	light wt anti
Native Light WT^e^	Parallel	light wt para
Transient Light WT^f^	Anti-parallel	tr light wt anti
Transient Dark WT^g^	Anti-parallel	tr dark wt anti
Transient Dark WT^h^	Parallel	tr dark wt para
Light G46S:G80R	Parallel	light g46s g80r para
Dark G46S:G80R	Parallel	dark g46s g80r para
Light V48I:G80R	Anti-parallel	light v48i g80r anti
Dark V48I:G80R	Anti-parallel	dark v48i g80r anti

^a^ ZTL structures investigated in the present study.

^b^ There are two different dimer structures: parallel and anti-parallel.

^c^ Abbreviations used in figures.

^d^ Native Light WT Anti-parallel structure was constructed from the Light V48I:G80R crystal structure.

^e^ Native Light WT Parallel structure was constructed from the G46S:G80R crystal structure.

^f^ Transient Light WT Anti-parallel was constructed from the Native Dark WT Anti-parallel structure by modeling the FMN in the light state.

^g^ Transient Dark WT Anti-parallel was constructed from the Native Light WT Anti-parallel structure by modeling the FMN in the dark state.

^h^ Transient Dark WT Parallel was constructed from the Native Light WT Parallel structure by modeling the FMN in the dark state.

### Role of Gln154 in the function of ZTL dimer

ZTL function within the circadian clock is dictated by daily changes in light intensity. The consensus model of LOV signaling involves a light-driven Gln-flip in response to changes in flavin N5 protonation.[11,16,17,24] In most LOV structures, the conserved Gln residue occupies a buried conformation coplanar to the flavin isoalloxazine ring. In the dark state, N5 is unprotonated, with the active site Gln forming H-bonds to the N5 and O4 positions via its amide functionality.[16,17,24] Upon illumination, N5 becomes protonated, leading to 180° rotation of the active site Gln to allow H-bond formation between the Gln-carbonyl group and N5-H.[16,17,24] The result is a disruption of contacts with residues in A*β* strand promoting conformational changes in N/C-terminal extensions to the LOV core.[11,16] Structures of ZTL indicate an alternative mechanism, where in the dark state Gln154 is dynamically adopting heterogeneous conformations between exposed (perpendicular to the isoalloxazine ring) and buried conformations. Light-activation was then predicted to drive rotation of Gln154 towards the buried conformation leading to downstream signal transduction.[16]

**Fig 2:**
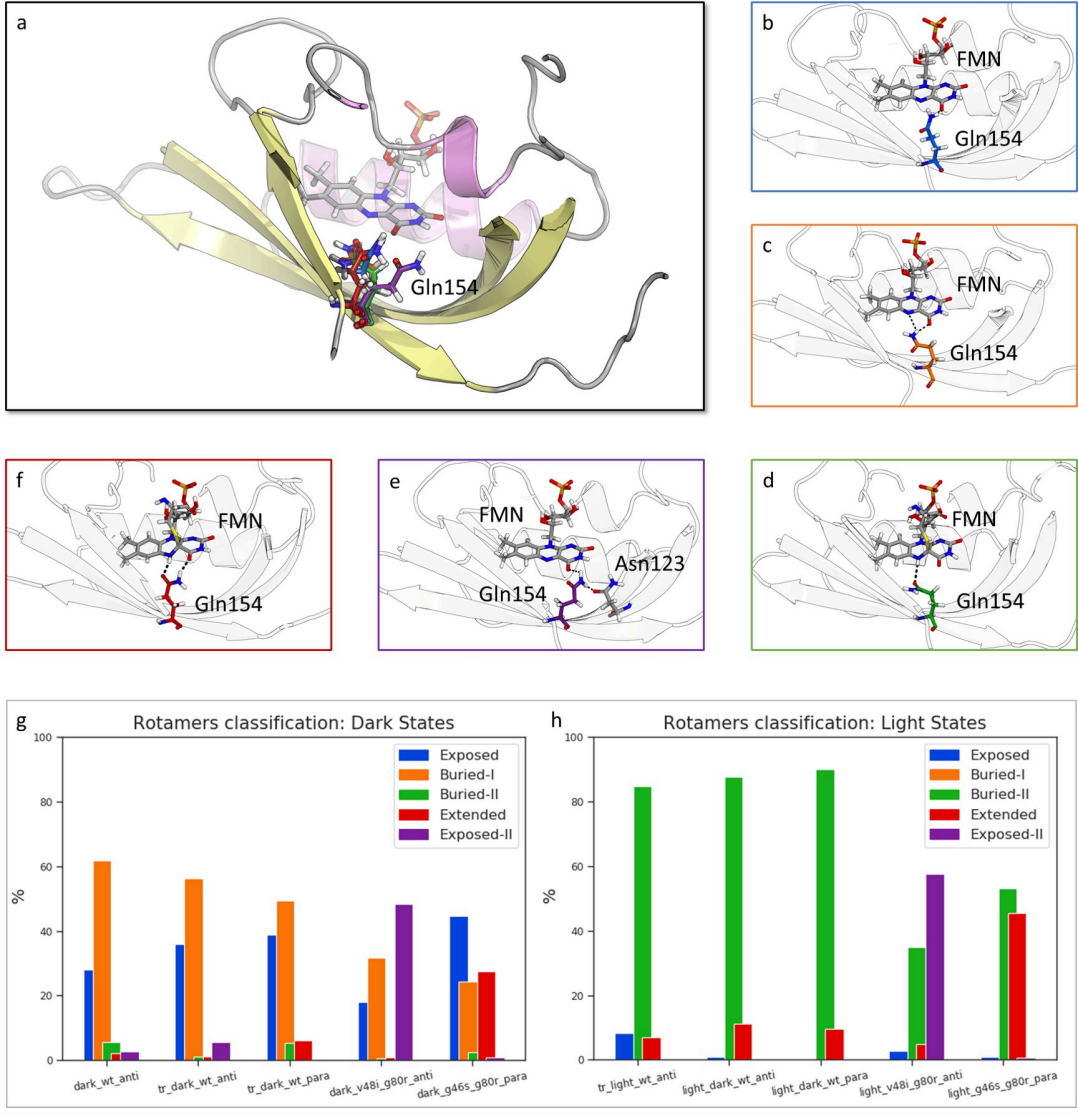
Conformational analysis of Gln154. a) Overlap of different Gln154 conformations. Representative Gln154 structures of b) Exposed conformation, c) Buried-I conformation, d) Buried-II conformation, e) Exposed-II conformation, f) Extended conformation. Hydrogen bonds formed by Gln154 shown in dashed line. g) Percentage of different conformations adopted by Gln154 during the simulations for ZTL structures in the dark state. h) Percentage of different conformations adopted by Gln154 during the simulations for ZTL structures in the light state.

To characterize the protein structural and dynamical profile related to the Gln154 switch, we focus on the analysis of Gln154 orientations corresponding to different FMN states in WT and allosteric variants. Based on the hydrogen bonds between Gln154 and FMN, we identified three main conformations: exposed, buried-I, and buried-II. The exposed conformation is identified when the only interaction presented between the FMN and Gln154 is a hydrogen bond between the side chain amino group and the O4 position of the FMN (Fig 2B). The buried-I conformation is formed when a hydrogen bond between the Gln154 side chain amino group and the N5 of the FMN is present (Fig 2C), in addition to the hydrogen bond in the exposed conformation. The buried-II conformation is formed when the carbonyl moiety of the Gln154 side chain forms a hydrogen bond with the protonated N5 of the FMN (Fig 2D). To quantify which conformations of Gln154 are sampled in different ZTL structures and states, we used a combination of the chi1, chi2, and chi3 dihedral angles (S4 Fig), which can uniquely identify different Gln154 conformations. The representative values for each conformation are provided in S1 Table.

To quantify which conformations Gln154 explores in different ZTL structures and states, we performed a similarity analysis. For each frame, the set of dihedrals are compared to the representative dihedrals of the Gln154 conformation described above. The conformation is assigned based on similarity (Fig 2G and 2H). Consistent with the WT structures, these three conformations differentiate dark- and light-state configurations of WT ZTL. Specifically, in the dark state, Gln154 is heterogeneous and samples both exposed and buried-I conformations with a preference for the buried-I conformation (Fig 2B and 2C). Photoactivation leads to a strong bias toward the buried-II conformation observed in the light-state ZTL and other LOV structures, supporting the ordering of Gln154 locus.

In contrast to WT MD simulations, exposed, buried-I, and buried-II conformations are insufficient to describe the conformational states of Gln154 in allosteric variants G46S:G80R and V48I:G80R. Specifically, in V48I:G80R a significantly exposed conformation (chi2 angle of approximately -177°; Exposed-II conformation) with an additional stabilizing H-bond formed with Asn123 is the predominant orientation (Figs 2E and S5). The exposed-II conformation is consistent with V48I:G80R structures, which are trapped in a predominantly dark-state conformation and functions as dominant-dark proteins *in vivo*.[16] In G46S:G80R, a different additional conformation is present, in which the amino group of the Gln154 side-chain forms a H-bond with the O4 position of FMN in addition to the H-bond in the buried-II conformation (Fig 2F). We refer to this latter state as an extended conformation.

The Gln conformational analysis provided insight into both the ZTL mechanism, and variances between Gln dynamics in MD simulations and the static structures. First, as noted above, dihedral-based conformation classification demonstrates a dynamic dark-state Gln154 conformation that flips between buried-I and exposed conformations. A dynamic dark-state Gln conformation is consistent with WT ZTL structures.[16,23]

Light-activation leads to Gln154 adopting a buried-II conformation for over 80% of the trajectories (Fig 2H), confirming light-driven ordering of Gln154 in a buried conformation. In contrast to WT proteins, G46S:G80R Gln conformational dynamics deviates from those observed in static crystal structures. Experimental studies showed that in G46S:G80R and G46A:G80R structures Gln154 largely occupied a single conformation where the Gln154 side chain was rotated toward the buried-I conformation. In contrast, MD simulations indicated that G46S:G80R could sample the exposed conformation, and could further sample a unique extended conformation containing H-bonds to both the N5 and O4 positions. These divergent observations can provide some insight into prior *in vitro* functional assays of G46S containing variants. Even though G46S variants demonstrate light-state like activity, exposure to light can still enhance light-driven complex formation with GIGANTEA.[23] These aspects can be explained by the Gln conformational analysis, specifically, the ability to form both Buried-II and Extended conformations. Both conformations result in Gln154 being largely co-planar with FMN, and prevent sampling of dark-state like exposed conformation leading to an enhanced light-state response.

Overall, these results complicate the Gln-flip mechanism. Although ZTL and LOV proteins retain a light-state signaling buried-II conformation, ZTL light-state activation largely results in ordering of the Gln154 side chain as opposed to a simple Gln-flip mechanism observed in other LOV proteins. Further, side chain identities at the G46 and V48 positions in A*β* strand can significantly perturb the Gln154 landscape to disrupt allosteric signal transduction. Although providing insight into the proximal light-driven signaling events, these observations lead to two conundrums regarding reconciling aspects of downstream signal transduction in ZTL: i) If Gln154 H-bonds are still driving Gln154 ordering and signal transduction, how do ZTL proteins signal in a Q154L background; ii) If G46S proteins are still sampling an exposed Gln conformation, why do they prefer the light-state parallel LOV dimer. To answer these two questions, we performed detailed analyses of Gln conformational effects on N/C-termini conformational changes dictating dimer reorganization and light/dark-functionality.

### Gln154 dynamics regulates downstream allosteric changes

#### C-terminal Conformational Changes and Gln154

Structural studies of ZTL allosteric variants identified two structural motifs coupled to Gln154 conformational dynamics. These consist of an N-terminal Cys45-Gly46-Phe47 (CGF motif) and a C-terminal Gln154-Phe155-Phe156 (QFF motif) that are predicted to couple Gln154 conformational changes to the Ncap and Ccap, respectively. In allosteric variants that trap a light-state-like conformation (G46S:G80R), rotation of Gln154 to the buried conformation is coupled to the movement of Phe156 towards the flavin binding pocket, and formation of a salt-bridge between Glu158 and Arg125.[23] Analogous conformational changes have been observed in LOV structures that contain a Gly residue at the position equivalent to Gly46, where a different conserved Phe residue (Phe66 in ZTL) undergoes light driven conformational changes coupled to Gln reorientation.[23,24,35] To identify the role of Phe residues in dictating signal transduction in ZTL, we examined Phe66 and Phe156 conformations in WT and variant ZTL trajectories.

**Fig 3:**
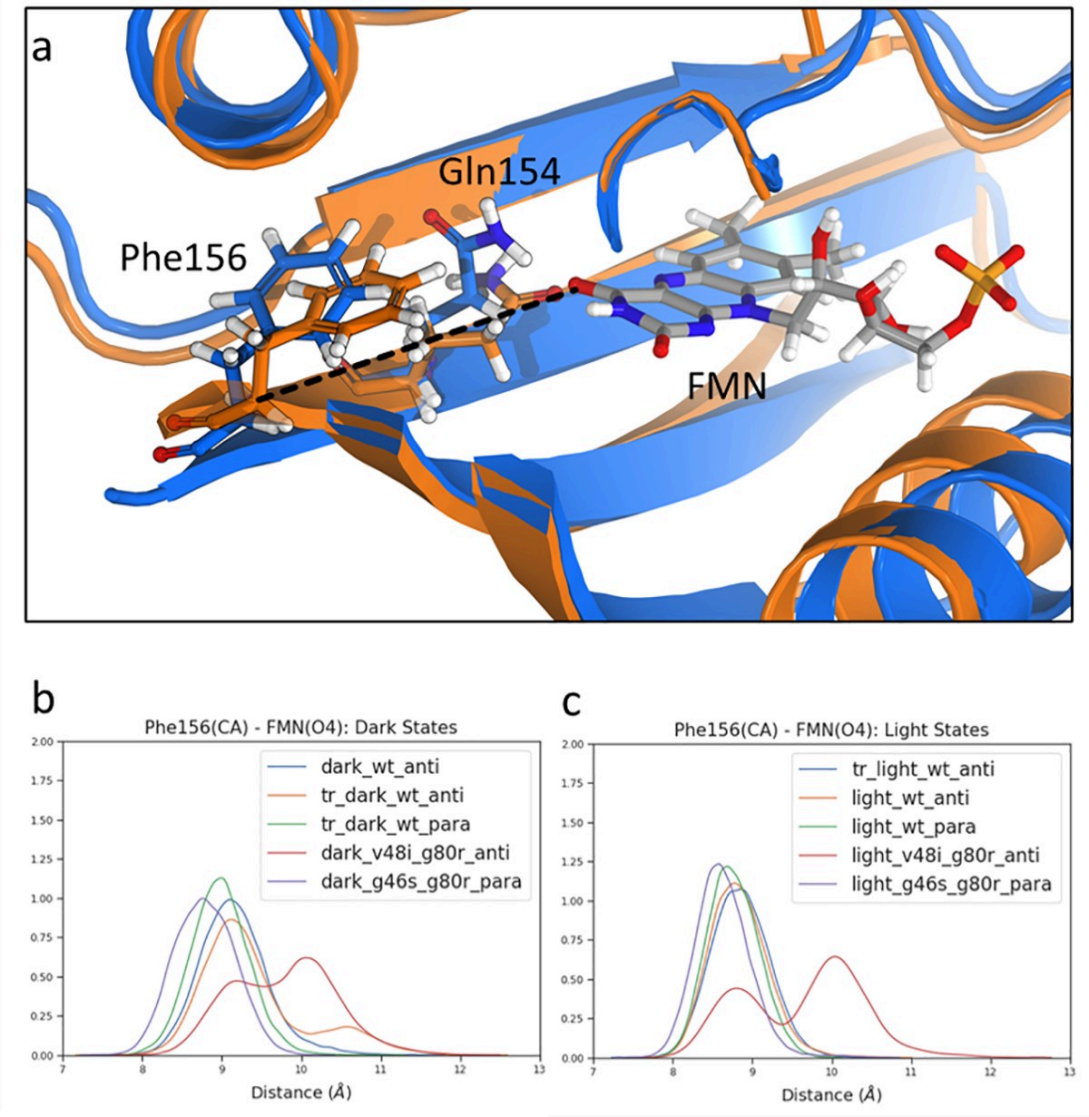
Light dependent Iβ strand pull investigation. a) Structure of the Iβ strand pull after Gln154 rotation. Dark state is represented in blue, and light state is represented in orange. Distance measured indicated by dashed line. b) Distribution of Phe156 CA and FMN(O4) distance for ZTL structures in the dark state. c) Distribution of Phe156 CA and FMN(O4) distance for ZTL structures in the light state.

Consistent with structures of ZTL allosteric variants, we observe that Gln154 rotation from exposed to buried-I and/or buried-II conformations is coupled to ordering (pulling) of the terminal part of I*β* strand and movement of Phe156 towards a cleft vacated by Gln154 rotation. The pull-effect has been quantified by monitoring the distance between the C*α* of Phe156 and the O4 of FMN (Fig 3A). Notably, the pull-effect is enhanced in WT light-state structures where Phe156 lies closer to the active site flavin (Fig 3B and 3C). Examination of the allosteric variants V48I:G80R and G46S:G80R confirms alteration in Phe156 movement, where V48I negates, and G46S enhances the pull-effect, consistent with dominant-dark and dominant-light state signaling respectively. These results verify that Phe156 movement towards the active site flavin is a proximal event dictating C-terminal conformational changes following adduct formation, and a determining factor in allosteric ZTL variants.

**Fig 4:**
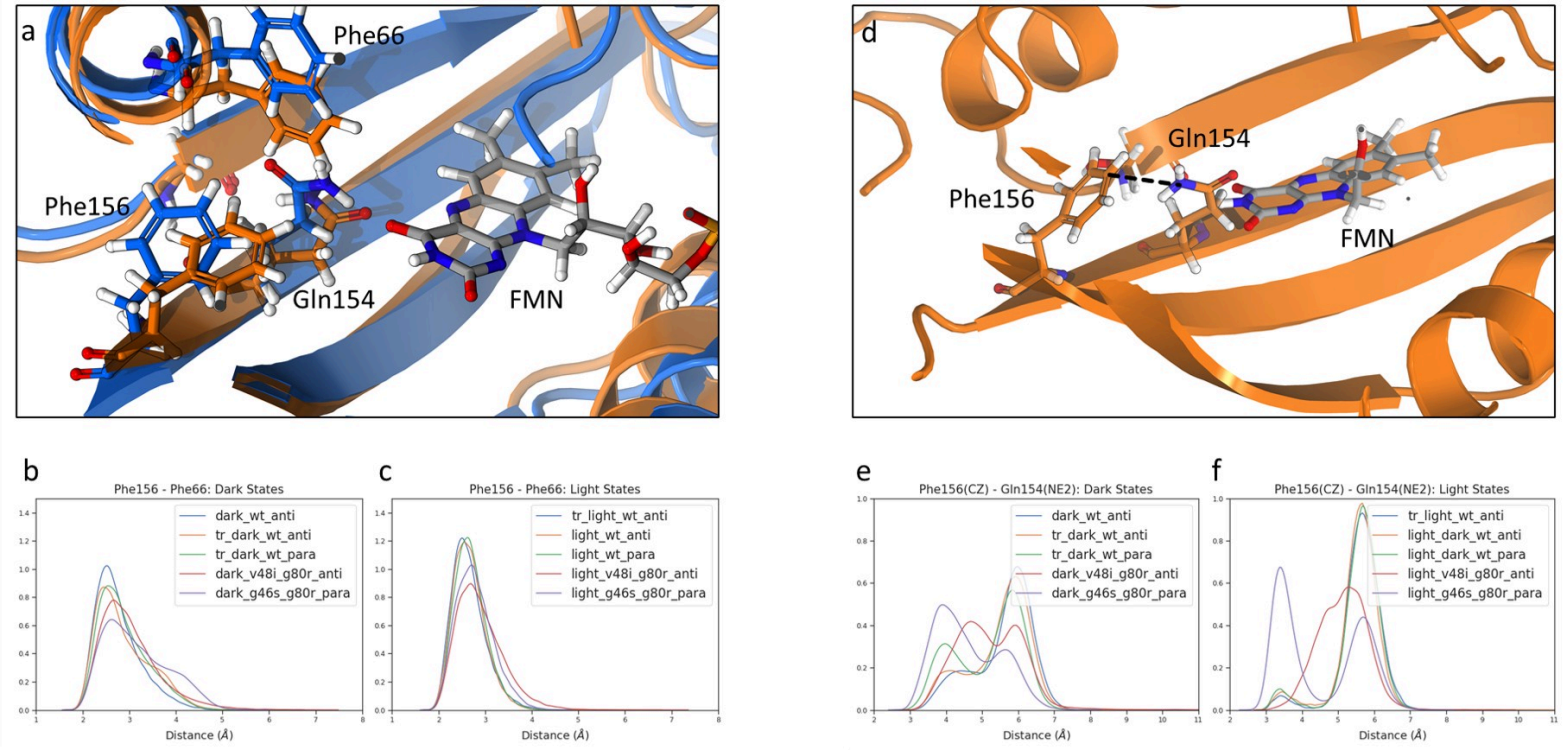
Phe156 stabilization factors for WT and G46S:G80R mutant. a) Structure of π-stacking interaction between Phe156 and Phe66 found in WT structures. Dark state is represented in blue, and light state is represented in orange. b) Distribution of Phe156 and Phe66 distance for ZTL structures in the dark state. c) Distribution of Phe156 and Phe66 distance for ZTL structures in the light state. d) Illustration of interaction between Phe156 CZ and Gln154 NE2 found in G46S:G80R structures. e) Distribution of Phe156 CZ and Gln154 NE2 distance for ZTL structures in the dark state. f) Distribution of Phe156 CZ and Gln154 NE2 distance for ZTL structures in the light state.

Previous structural studies suggested that movement of Phe156 may be stabilized by a *π*-stacking interaction between Phe66 and Phe156.[16,23] Further, the residue equivalent to Phe66 undergoes light-driven conformational changes in LOV structures containing a Gly at position 46.[24,35] To further understand the role of Phe residues in LOV signal transduction, we examined Phe66 dynamics in WT and variant ZTL structures. The concerted movement of Phe156 and Gln154 rotation was assessed and quantified using the Pearson Correlation (PC) coefficient (S2 Table). In ZTL, we observe significant movements of Phe156 and Phe66 towards the FMN binding site that is coupled to Gln154 rotation. Movement of Phe66 mirrors analogous light-driven conformational changes in *Bacillus subtilis* (YtvA) and a short LOV protein in *Pseudomonas putida* (PpLOV) that contain Gly residues at positions equivalent to Gly46.[24,35] In ZTL, movement of Phe66 and Phe156 as a consequence of Gln154 rotation produces a *π*-stacking interaction that is light-state selective (Fig 4B and 4C), where we observe a narrow spread and higher density of the distributions in the light-state structures.

**Fig 5:**
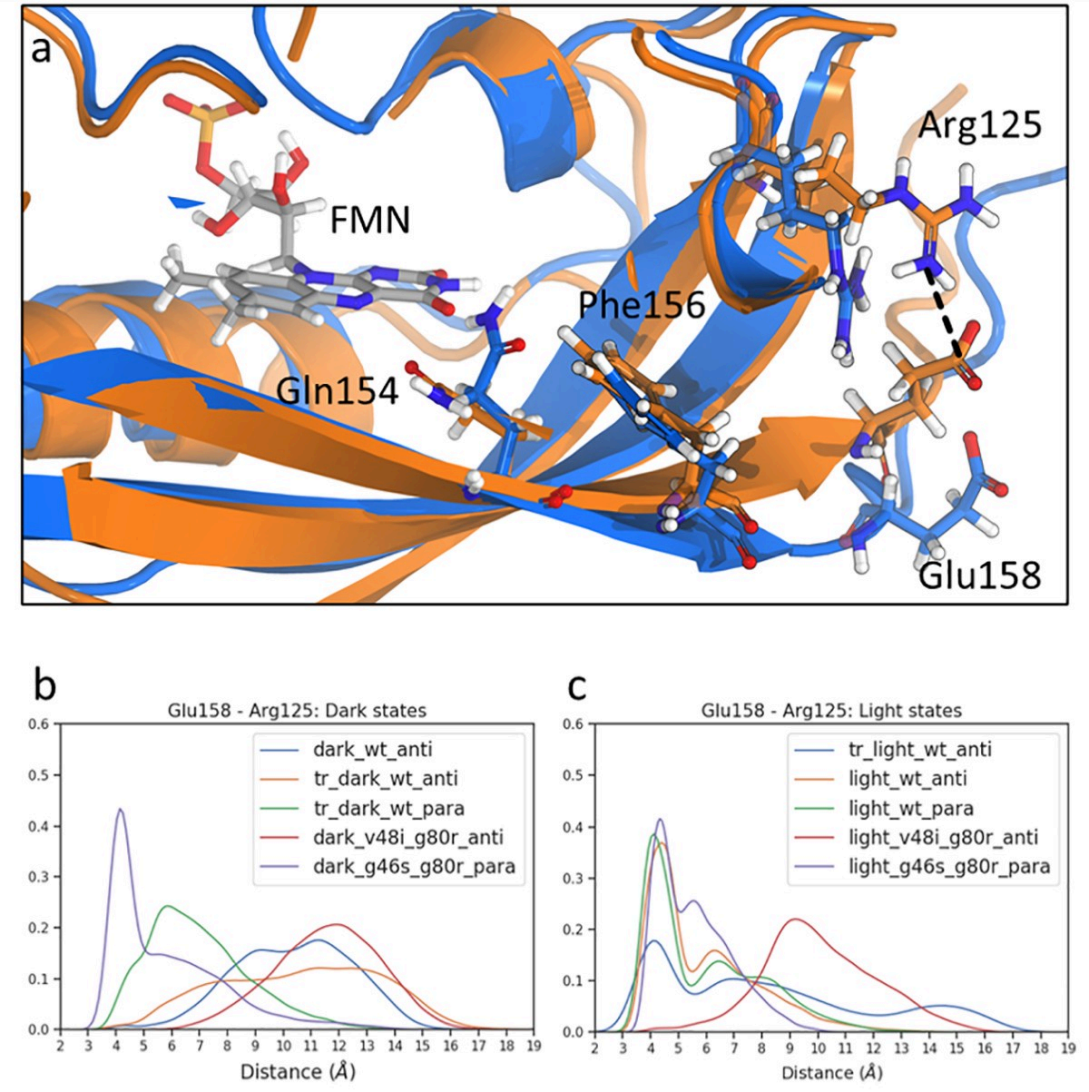
Salt bridge between Glu158 and Arg125. a) Structure of salt bridge between Glu158 and Arg125. Dark state is represented in blue, and light state is represented in orange. Distance measured represented in dashed line. b) Distribution of Glu158 and Arg125 distance for ZTL structures in the dark state. c) Distribution of Glu158 and Arg125 distance for ZTL structures in the light state.

Examination of allosteric variants V48I:G80R and G46S:G80R demonstrates perturbation of Phe156 and Phe66 movement. As expected, introduction of V48I sterically hinders Gln154 rotation (S6A Fig) and prevents Phe156 movement, consistent with locking ZTL into a dark-functional state. Because the dark-state of G46S:G80R mutant mimics the light state conformation, it is expected that this state also favors the light-state-like *π*-stacking interaction between Phe66 and Phe156. However, this is not the case as the dark-state of G46S:G80R mutant displays the lowest peak of the Phe66 and Phe156 distance distribution among all dark states (Fig 4B). According to the pull of the Phe156 (Fig 3) and the consequent salt bridge at the C-terminal (Fig 5), the light-state-like allosteric perturbation is still present in the G46S:G80R mutant. Since the *π*-stacking between Phe66 and Phe156 seems not to be a key factor to stabilize Phe156 in the pocket, an alternative interaction should take place for this purpose. Examination of distances between Gln154 NE2 and Phe156 CZ highlights an alternative allosteric route coupling Gln154 and Phe156 movement. A Coulomb interaction between the partial positive charge of the amino group and the negative charge of the aromatic ring couples Gln154 and Phe156 resulting in characteristic short distance peaks in both dark- and light-states of G46S:G80R (Fig 4E and 4F). In this manner, electrostatic interactions drive a light-state like “in” conformation of Phe156 regardless of light-driven *π*-stacking interaction with Phe66.

Crystal structures of the allosteric variant G46S:G80R indicate that Phe156 movement may be coupled to Arg125-Glu158 salt bridge formation.[16,23] However, whether this salt-bridge formation was an artifact of crystal packing interactions, or mainly due to the parallel dimer orientation of G46S:G80R, or unique to the G46S allosteric variant was unknown. To further reveal the light-driven conformational changes between the proximal CGF motif and the Ccap, we analyzed the correlation between FMN light-states and formation of the Arg125-Glu158 salt bridge in all WT and variant trajectories. In the WT dark-state, as well as both dark- and light-states V48I:G80R trajectories, the C-termini were free to fluctuate and exhibited Arg125-Glu158 distances incompatible with salt-bridge formation (Fig 5B). In contrast, WT light-state, as well as both dark- and light-states G46S:G80R, trajectories exhibited a sharp peak around 4 Å consistent with a stable salt bridge between these two residues (Fig 5C). The distance analysis reveals the coupling of conformational changes in the QFF motif to long-range conformational changes following adduct formation in WT proteins. These confirm that the effects of allosteric variants at the G46 (light-state like) and V48 position (dark-state like) propagate to signaling elements within the Ccap.

Overall, analysis of putative signaling elements within the proximal QFF motif and downstream Ccap elements suggests a concerted mechanism coupling Gln154 dynamics to ordering of the C-termini. For all variants and light-states, salt-bridge formation directly correlates with adduct formation, Gln154 ordering, and movement of Phe66 and Phe156 to form stabilizing *π*-stacking interactions. These conformational changes are dependent upon the unique presence of a Phe residue at position 156, and delineates a mechanistic route at an atomistic level.

#### N-terminal Conformational Changes and Gln154

In addition to conformational changes within the Ccap, critical structural changes for light-dependent ZTL function occur at the Ncap. Original models of ZTL signaling relied on a shift in population of Gln154 from a heterogenous, but predominantly exposed, conformation in the dark state to an ordered buried conformation in the light state.[16] Although the above analysis shows a heterogeneous population in the dark, the buried-I conformation still dominates, and both the buried-I and buried-II conformations show the same degree of rotation around the chi2 angle (S1 Table). Thus, Gln154 orientation alone appears to be a poor choice of driving structural change at the Ncap. Further, given that G46S variants can still sample both buried-I and exposed conformations, what factors dictate the observed light-state like allosteric effect at the Ncap are poorly understood. To alleviate these discrepancies, we examined dark- and light-state trajectories to identify additional interactions that differentiate buried-I, buried-II, and exposed conformations.

**Fig 6:**
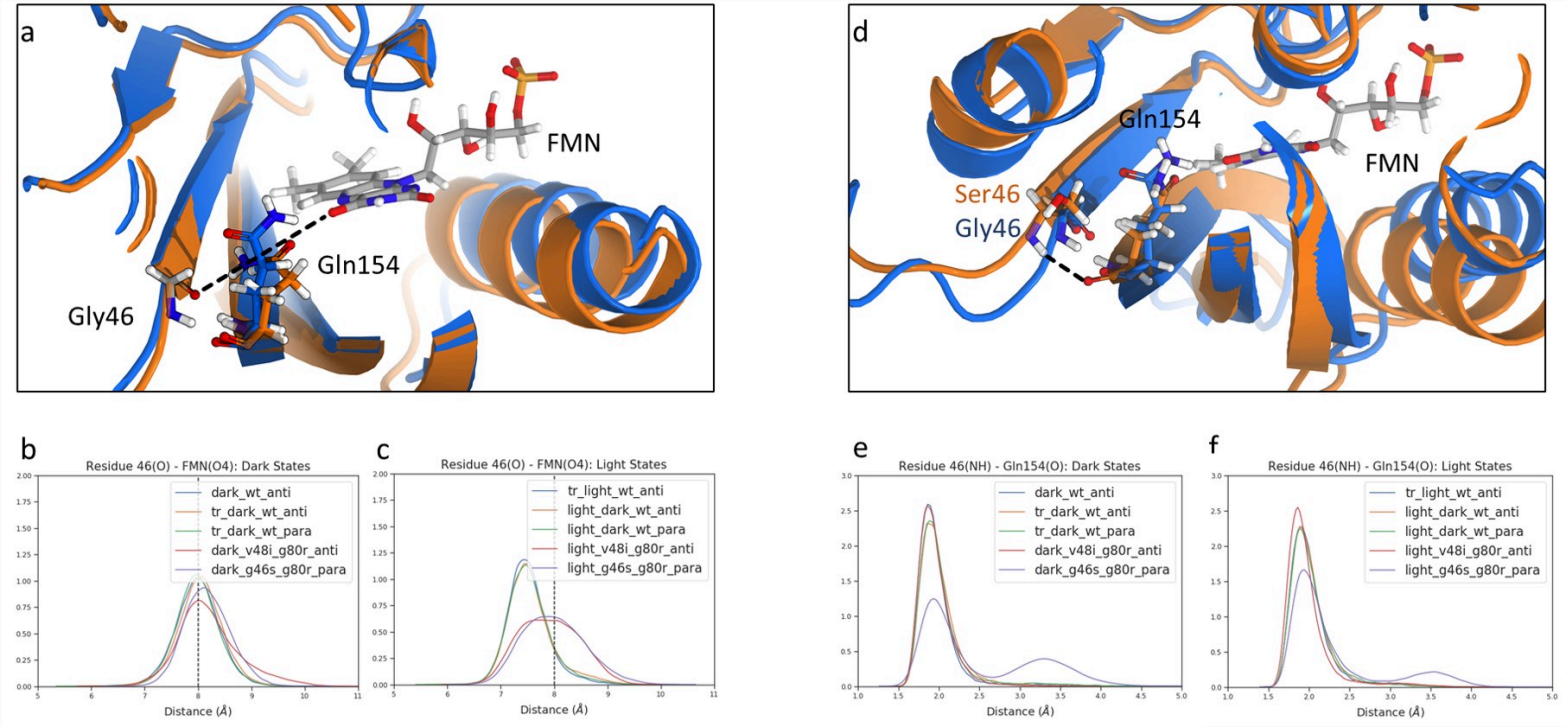
Routes of N-termini perturbation in ZTL. a) Hydrogen bond between Gln154 NE2 and Gly46 O responsible of N-termini perturbation in WT structures. Dark and light states representative structures are in blue and orange, respectively. Distance measured is represented in dashed line. Distributions of the distance between O of residue 46 and FMN O4 for ZTL structures in b) the dark state and c) the light state. d) Illustration of the perturbation at the N-termini created by Ser46 in G46S:G80R (orange) compared to a WT structure (blue). Distributions of the distance between NH of residue 46 and O of Gln154 for ZTL structures in e) the dark state and f) the light state.

Our analysis indicates that in the buried-II conformation, the amino group of the Gln154 side-chain can form an H-bond with the proximal Gly46 carbonyl moiety, which is located at the interface between A*β* and the Ncap (Fig 6A). This interaction results in a “pulling” effect on Gly46 specific to the light-state buried-II conformation (Fig 6B and 6C). The “pulling” effect observed in WT ZTL structures is analogous to allosteric mechanisms in the fungal circadian clock protein VVD,[11] where the Gln-flip (buried-I to buried-II) permits formation of an H-bond between the active site Gln and the carbonyl of Ala72 and pulling of the Ncap towards the LOV core. Importantly, Ala72 structurally occupies the same site in A*β* strand, indicating conservation of a signaling motif in fungal and plant circadian clock proteins.

Notably, V48I:G80R and G46S:G80R allosteric variants are exceptions to this mechanism. The first is expected, as it prevents the Gln154 from switching to a buried conformation via steric hindrance that functionally locks ZTL in a dark-state-like conformation. For the latter, a more in-depth investigation is necessary to explain how this mutant is able to cause a light-state functional perturbation within the Ncap. Examination of G46S:G80R structures and trajectories identifies an alternative allosteric route, in which the steric presence of the Ser46 side chain alters the distance distribution between the Ncap and Gln154 leading to an increase in the distance of Ser46 from the LOV core (Fig 6D). From these structural analyses, we delineate two alternative routes for propagation of allosteric effect following light-activation.

We observed that in the WT, light activation first results in ordering of Gln154 in a buried-II conformation, leading to the formation of: i) *π*-stacking interactions between Phe66 and Phe156, which is responsible for propagating the structural change to the Ccap, ii) H-bond between Gln154 and Gly46, which is responsible for propagating conformational changes to the N-termini. This WT allosteric mechanism is altered in the G46S:G80R variant where the presence of Ser46 induces: i) a unique extended Gln154 conformation, which stabilizes Phe156 in the FMN binding pocket via electronic interactions resulting in propagating a structural change to the Ccap, and ii) a Gln154 conformation independent, steric-based conformational change at the ZTL N-termini.

### Gln154 effect on ZTL conformational flexibility

The investigation of the structural changes in the N- and C-termini granted only a partial picture of the possible downstream effects of ZTL light-dependent Gln154 conformational gating. In fact, shifts in the population distributions on the conformational landscape without appreciable structural transformations can contribute to the allosteric protein functional changes.[36–49] To show how alteration in Gln154 conformational sampling in WT ZTL and allosteric variants impacts the ZTL structural landscape, we performed time-structure Independent Component analysis (tICA) by featurization of the protein conformation using all the C*α* pairwise distances. The resulting 2-dimensional plots represent the impact of light- and dark-states and allosteric variants on the ZTL conformational space explored.

**Fig 7:**
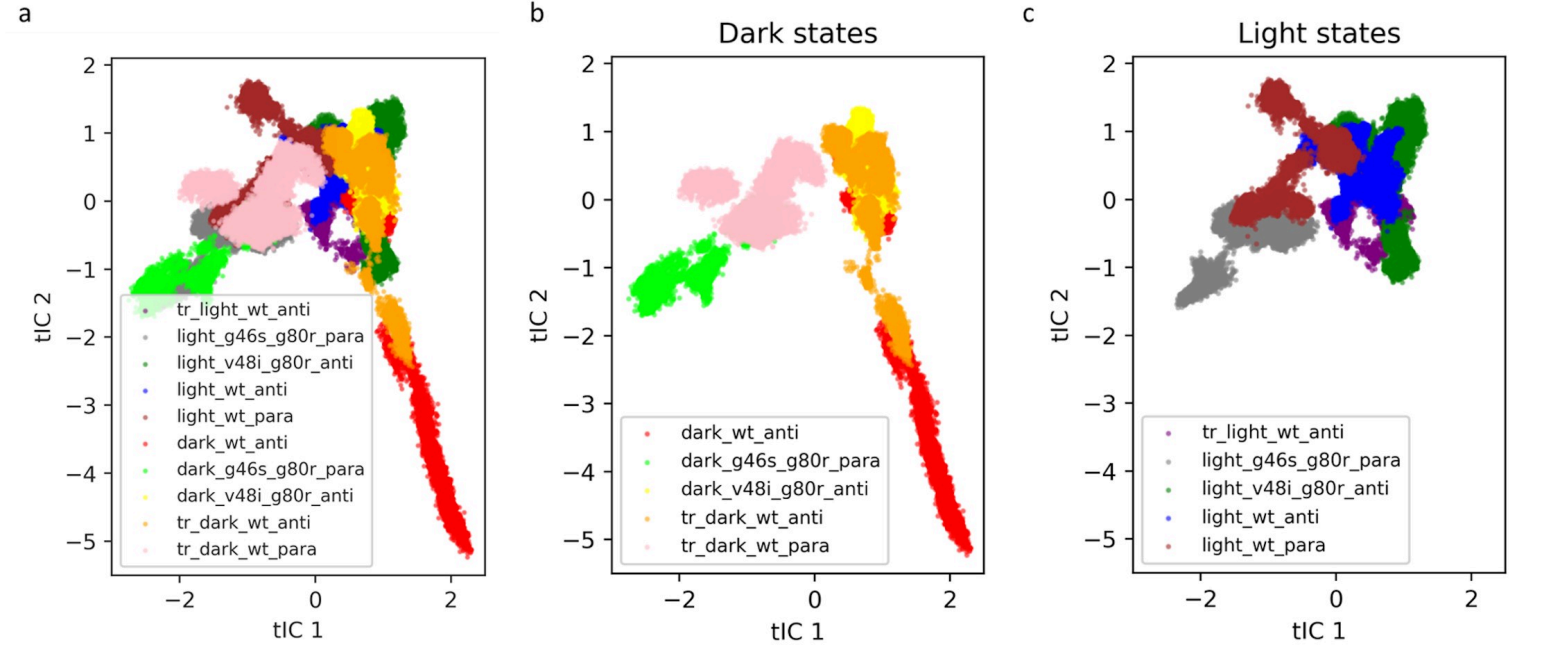
Dimensionality reduction analysis using tICA method. a) All structures. b) Dark states including the dark native anti-parallel and the transient dark states. c) Light states including the light native parallel and the transient light states.

The two components corresponding to the slowest-relaxing degrees of freedom, which associate with functionally relevant motions, distinguish the native dark- and light-state structures (Fig 7). Specifically, the native dark-state (Red, dark_wt_anti), samples considerably more conformational space than the native light-state (Maroon, light_wt_para), consistent with ordering following adduct formation (Fig 7A). To verify the effect of adduct formation and Gln154 conformation on ZTL dynamics, we further analyzed the conformational space explored in the transient structures, which mimics the initial landscape sampling immediately following adduct formation (transient-light) and scission (transient-dark). Consistent with disorder-order transitions, the transient dark state (tr_dark_wt_anti) stretches from the light state region to the dark native region. Further, the transient light state (tr_light_wt_anti in Fig 7C) displays dynamical behavior similar to the light state. This is indicated by its overlapping with the native light state structures in the tICA plot and by significantly less conformational space explored. These results confirm that light drives a reversible disorder-order transition that is directly coupled to adduct formation and Gln154 dynamics (Fig 7).

Examination of how the conformational landscape is perturbed by G46 and V48 allosteric variants provides insight into how subtle local alteration in side-chain identity can impact dynamics-driven allostery. Specifically, the conformational space sampled by the V48I:G80R mutant in the dark state is more restricted than the WT-dark state and largely overlaps with the anti-parallel transient states (dark_v48i_g80r in Fig 7). These results are consistent with restricting the Gln154 conformation to a primarily exposed conformation. Similarly, the light-state conformations are more restricted, leading to only minimal overlap between V48I:G80R and the native light parallel simulations (Fig 7C). Thus, introduction of V48I restricts dynamic motions gating interconversion between light- and dark-configurations. In contrast, G46S variants lock the dynamics away from the dark state structures (dark_g46s_g80r and light_g46s_g80r in Fig 7), supporting light-state-like functionality in the absence of the flavin C4a adduct.

In summary, tICA was used to analyze the effect of mutations and light state on the overall protein dynamics. In particular, the dark state displays higher flexibility, while the light state seems to have limited protein motions. Although the tICA plots show overlap between parallel and anti-parallel structures, it is important to point out that in our simulations the inter-conversion between the parallel and anti-parallel dimers was not fully observed. Rather, this analysis captured dynamic similarities between different systems, which were strongly influenced by monomeric atomic motions (S7 Fig). We observed a correlation between the protein flexibility deduced by the tICA projections and the Gln154 conformational dynamics. This indicates the relationship between Gln154 and the protein dynamics, whereby an ability to interconvert between Gln154 conformations is essential to drive light-dependent allosteric conversions in ZTL structure. To better understand how these dynamic motions kinetically gate conformational changes we exploited the reduced dimensional space generated using the tICA to generate a Markov state model.

### Identification of functional stable states in ZTL conformational landscape

Markov State Model (MSM) can help to discretize the protein conformational landscape into functional metastable states and obtain a kinetically relevant picture of ZTL allosteric process. To achieve this goal, we followed the following protocol: i) k-means micro-clustering into microstates, ii) building of a MSM, and iii) Perron-cluster cluster analysis (PCCA) for transition-based macro-clustering. The details for each step can be found in the Materials and Methods section.

**Fig 8:**
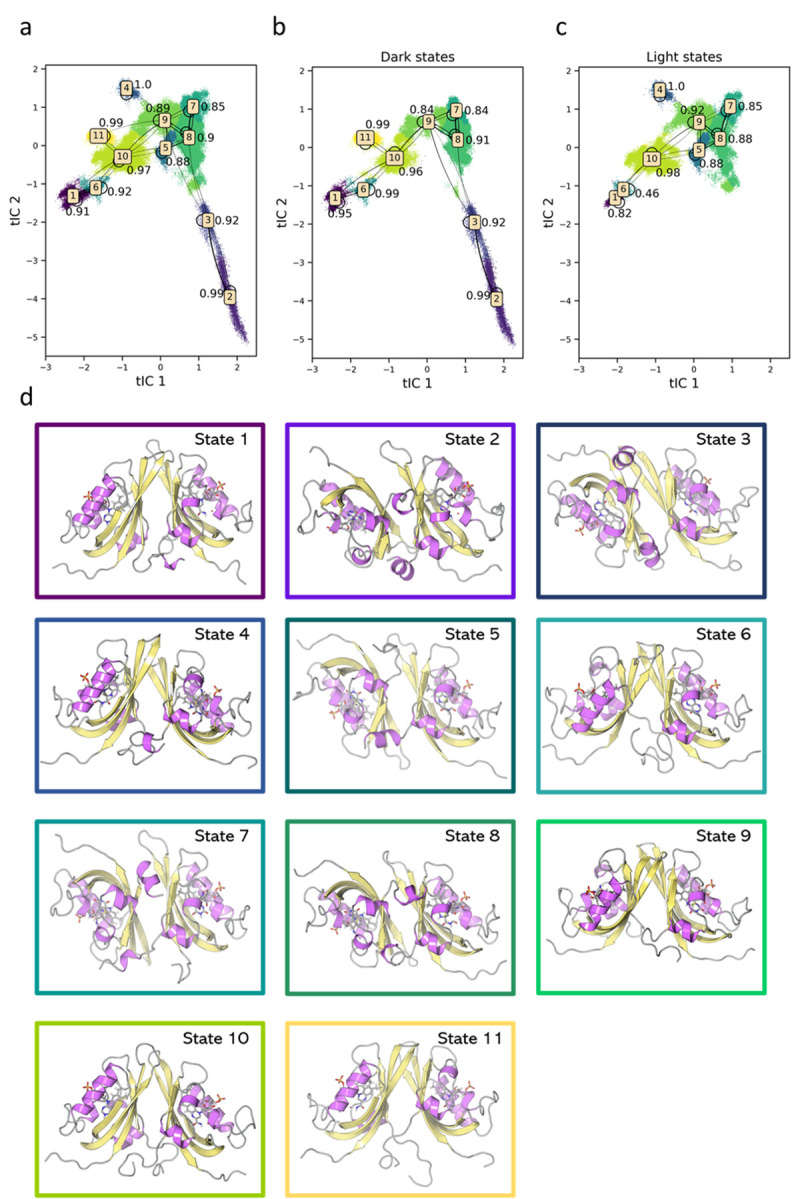
Transition probabilities among different macrostates in the Markov state model. a) Transition probabilities among macrostates using all trajectories; b) Transition probabilities based on Dark state trajectories; c) Transition probabilities based on Light state trajectories; d) Representative structures for different macrostates obtained from each cluster center.

Different metastable states lie in different free energy basins illustrating the effectiveness of PCCA in separating kinetically separated states (S8B Fig). As shown in Fig 8, the probability of a structure to remain in its original state is higher than the probability for it to transition to other states. This indicates that each macrostate is in a minimum on the free energy surface, and that the kinetic barriers prevent the system from switching among macrostates frequently. The occupation of these macrostates from different ZTL simulations is plotted in S9A Fig. This analysis allowed the division of the projected conformational space into four areas: Dark Native, Light Native, Transient and Transient Parallel (S9B Fig), based on the structures that predominantly occupy each macrostate. The effect of the light-state adduct on the protein transitions is investigated by plotting the transition for light and dark structures separately (Fig 8B and 8C).

The plot of the transition probabilities among different metastable states (Fig 8) shows that the states contained in the transient area (States 3, 5, 7, 8 and 9) have higher probability to transit to the states occupied by the native conformations. This confirms the role of the transient states as intermediates between the stable native dark and native light states.

When the FMN cofactor is modeled in the light state, the conformations of the protein shift from dark state-like State 2 to the intermediates between the State 2 and the light-state like State 5. This shift of conformations mimics the biological process of the protein changing its functional dynamics and structures upon change of light conditions.[13,15]

As shown in Fig 8B, the native dark state structures cover a large area, exploring most metastable states except for States 4 and 5. This shows that the absence of the photo-induced covalent bond between FMN-Cys82 correlates with the high conformational flexibility. On the contrary, the distribution of the light states is limited in a narrow area (Fig 8C), suggesting that the photo-induced covalent bond between FMN-Cys82 correlates with a low conformational flexibility.

Fig 8D illustrates representative structures for the 11 metastable states identified in the MSM. Interestingly, there is a partial rotation of the two monomers in State 11, suggesting a rotation of the monomers towards the anti-parallel conformation. This state is populated solely by parallel transition dark structures, whose RMSD was found to significantly fluctuate during the MD simulations (Protein Stability Analysis in S1 Text). The combination of these two observations could provide an ulterior dynamical confirmation of the light influence to ZTL dimer orientation.

However, from the visual inspection, we were not able to find other clear structural differences among different metastable states. For this reason, we employed a machine learning based classification model to identify other structural differences that may play a significant role in ZTL allostery.

### ZTL allosteric structural changes captured by Machine Learning Modeling

In order to gain further structural insight into ZTL allostery, a machine learning (ML) model was employed to identify non-trivial structural changes among protein metastable states. This model is carried out on the entirety of the data generated using the MD simulations, and depicts a dynamical nature in the structural characterization of ZTL allosteric process. S10 Fig shows a validation of our method in discerning high importance structural features.

Through this ML model, it is assumed that the structural features with high importance are likely coupled with free energy barriers dividing macrostates and thus provide an atomistic insight into the allosteric mechanism associated with the identified macrostates. In this ML model, the features constructed based on the protein structure, in this case all the C*α* pairwise distances, are ranked using a percentage score based on their contribution in differentiating macrostates. Overall, the OvO-random forest classification model reached a validation accuracy 95% with 10 trees of 4 layers depth. 357 out of 33670 C*α* pairwise distances account for 90% of the distinguishability among macrostates (S11 Fig).

#### Structural effects of FMN states

Despite the fact that in our simulations we do not observe the complete interconversion between anti-parallel and parallel dimer conformations, it will be informative to find key conformational changes related to functional monomeric motions. To investigate structural changes not localized in the proximity of FMN binding site that arise in response to the changes in the FMN state, we applied ML analysis to key metastable states identified in the MSM. Of particular interest are the metastable states occupied mainly by the WT dark or light structures, and the transient states adjacent to them. The changes between the dark state and transient states were inspected by analyzing the most important structural differences that distinguish the State 2 as the dark native structure and State 3 representing a transient state adjacent to the State 2 (Fig 8D). We then compared States 3 and 9, as a mean to compare transient metastable states adjacent to the native dark and light states, respectively. Finally, State 9 and State 4 as the light native structure were compared.

**Fig 9:**
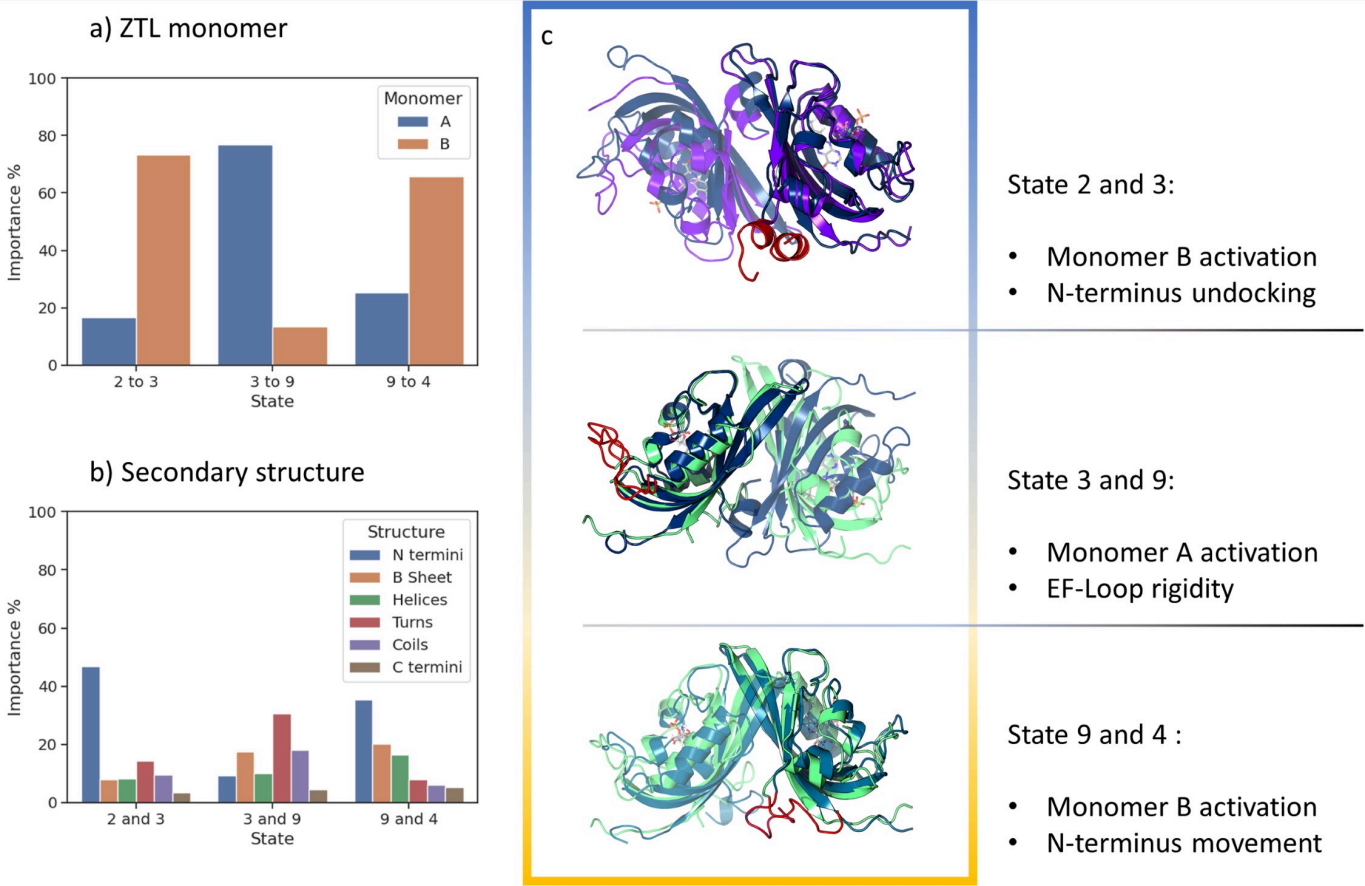
Structural changes among ZTL metastable states. a) Contribution from each ZTL monomer; b) Contribution from secondary structure. c) Illustration of important structural changes, highlighted in red, for each allosteric step.

Examination of these macrostates revealed coupling of key regulatory regions in ZTL. The ML classification model indicates that States 2 and 3 are distinguished from each other by undocking of the N-termini from the LOV-core and an increase in N-terminal rigidity (Figs 9B and S12B). The ML investigation of the structural differences between States 3 and 9 identifies the EF-loop as key to allosteric transitions. The EF loop is of unknown function and is only present in members of the ZTL-family and homologs of the fungal circadian clock photoreceptor VVD. In VVD, the EF-loop was presumed to be involved in accommodation of an FAD vs. FMN cofactor. However, such an explanation is not applicable to ZTL, which binds FMN. Rather, the EF-loop has been suggested to be involved in signaling by an unexplained mechanism. Here, ML classification models indicates that an increase in EF-loop rigidity is an essential structural feature to differentiate States 3 and 9 (Figs 9B and S12D). Examination of the light State 4 and adjacent transient State 9 indicates that the key structural feature that distinguishes these two states is an increase in rigidity of the N-termini, which moves closer to the LOV core (Figs 9B and S12F).

Overall, in the structural characterization of ZTL allostery, changes in the structural features within the monomers are dominant (S13 Fig). Furthermore, an asymmetric change of the two monomers was identified among the key metastable states investigated (Fig 9A). This may allow for the increased sensitivity to light-intensities as the activation of one monomer may be sufficient to activate the allosteric process in an asymmetric manner.

It is important to note that the overlap in the tICA space is due to the monomeric correlated motions of the anti-parallel and parallel dimers as the full transition between them was not observed (S7 Fig). Further sampling in the transition area between anti-parallel and parallel dimer conformations could reveal yet unexplored regions of the free-energy landscape that could reveal additional key conformational changes that are potentially involved in the monomers’ re-orientation.

#### Structural effect of ZTL variants

Comparison of the ML classification model with structural states occupied by allosteric variants provides further clarity on how mutations at the G46 and V48 positions alter dynamics driven allostery.

First, introduction of V48I constrains Gln154 in an exposed conformation impacting dynamics at the N/C-termini, rigidifying these regulatory regions in dark-state conformations (State 7; Fig 8). Comparisons to features identified in the ML classification model indicates that rigidification of the N/C-termini is coupled to rigidification of the E-F loop (S14 and S16 Figs). Thus, V48I variants restrict Gln154 dynamics, leading to a decrease in dynamics at key structural features necessary to light-dark transitions.

Regarding the G46S mutation, the serine induces a unique Gln154 extended conformation, which enhances the light-state activity by preventing the exploration of the dark state exposed conformation. This mutant is shown to initiate the perturbation to the N-termini using an alternative mechanism where the Aβ strand is pushed further from the LOV core. The ML classification analysis identifies that as a result of this alternative route of perturbation, N-termini remain far from the dimer interface in this variant, while in the wild type it crawls into the dimer interface (S15 and S17 Figs).

Overall, examination of the allosteric variants indicates two modes of allosteric perturbation. In V48I variants, the allosteric mechanism is not altered, rather V48I variants alter the dynamic dark-state landscape essential to ZTL allostery. Specifically, restriction of conformational freedom can trap the protein in dark-state-like configurations. In contrast, variants at the nearby G46 position can directly alter the allosteric mechanism to obtain structural changes in the functional N-termini as an effect of the steric hindrance caused by Ser46.

## Discussion and conclusion

LOV proteins are ubiquitous in nature and have been employed in optogenetic tools to impart photodynamic control over signaling domains. Due to their widespread utility, LOV proteins have been a central focus in both structural and computational approaches to delineate allosteric mechanisms. Currently, the consensus mechanism in LOV signal transduction necessitates the presence of a Gln residue adjacent to the O4-N5 positions of the flavin cofactor, where changes in N5 protonation can drive a flip in the Gln side chain. Recent structural studies of WT ZTL and allosteric variants identified an alternative signaling mechanism that can persist in the absence of a Gln residue, whereby in WT proteins the Gln rotates from a heterogeneous exposed conformation in the dark to a buried light-state conformation. In these studies, it was proposed that conserved Phe residues may be able to sense electronic changes in the FMN cofactor and mediate a Gln-independent signaling mechanism. Notably, light-dependent movement of Phe residues has been observed in structural and computational studies of LOV proteins that contain Gly residues at the position equivalent to ZTL G46. Despite observation of analogous processes in other LOV proteins, whether the structures observed in allosteric variants represent the ZTL signaling mechanism remained unproven. Further, how Gln conformational dynamics alter allosteric responses remained unexplored. In the present study we have performed detailed atomistic simulations, MSM analyses, and ML classification models to delineate allosteric effects that involve the entirety of the ZTL dimer structure. The results provide a unique insight into the ZTL allosteric mechanism that has impacts on the broader LOV community, as well as providing atomistic details on how different allosteric variants can perturb signaling through regulation of ZTL dynamics. We briefly discuss the consequences of these studies below.

### Proximal signaling event and a Gln-less mechanism

First, the computational tools employed herein verify proximal allosteric events identified in structural studies of ZTL allosteric variants. Namely, in the dark-state, Gln154 samples heterogeneous conformations that permit a dynamic dark-state landscape. Adduct formation triggers organization of Gln154 in a predominantly buried-II conformation that is coupled to the concerted movement of Phe66 and Phe156 stabilized by *π*-bond formation, and a new H-bond between Gln154 and Gly46.[50] The net result is ordering of the N/C-termini and formation of a light-dependent salt-bridge between Arg125-Glu158. We note that the observed allosteric process is directly analogous to recent computational studies of YtvA,[24] where they observed coupling between Gln conformational dynamics and light-dependent movement of a Phe residue equivalent to Phe66 (Phe46 in YtvA). These observations raise the possibility of a Gln-independent signaling mechanism that relies on Phe movement in response to changes in flavin electronics. The experimental studies conducted by Pudasaini et al[23] and Pérez et al[51] support this hypothesis by showing the ability of ZTL and YtvA to signal in absence of the Gln154.

To verify such a mechanism, we examined MD simulations of Q154L variants to determine whether Phe movement independent of Gln154 can mimic the ZTL allosteric response. Indeed, a preliminary analysis showed that in Q154L variants the adduct formation was coupled to the movement of Phe156 towards flavin O4 position in a manner analogous to the photoactivation mechanism of WT ZTL (S18 Fig). The reason for this movement can be identified by the interaction between Phe66 and FMN, observed via distance analysis in S19 Fig, which stabilizes Phe156. Thus, the Phe156 movement alone is sufficient for signaling and can persist in the absence of Gln154. We propose that a similar electronics-based signaling mechanism can occur in other LOV proteins containing Gly residues at positions equivalent to G46. Importantly, Gln➔Ala variants retain some light-regulated activity in YtvA, consistent with an electronics-based mechanism.[51]

### Allosteric response

Structural studies of WT ZTL and allosteric variants present a snapshot of ZTL in various functional states. However, they cannot provide insight into how local conformational changes propagate to a global rearrangement in a dimer interface. Further, traditional MD approaches alone are insufficient to capture a long time-scale reorganization of protein interactions (e.g. dimer interface). Here, we coupled traditional MD simulations in diverse functional states, and transient intermediates with MSM and ML classification models to capture how atomistic proximal events dictate dynamics of signaling motifs gating a conformational response. These approaches provide detailed information on the ZTL signaling mechanism and highlight structural motifs previously of unknown function.

MSM and ML classification models indicate that proximal structural changes within the CGF (N-terminal) and QFF (C-terminal) motifs dictate ordering of N- and C-termini. Central to N/C-termini ordering is formation of a salt-bridge between Arg125-Glu158 connecting the C-terminus to the helical LOV domain surface. ML classification models indicate that a central component of ZTL signaling and conversion between anti-parallel and parallel configurations is ordering of the EF-loop. Previous low-resolution studies of the ZTL family member flavin-binding, KELCH REPEAT, F-BOX 1 (FKF1), had identified the EF-loop as involved in light-dependent dimer reorganization via an unknown mechanism. Here, we demonstrate for the first time direct coupling of adduct formation to allosteric events within the EF-loop. Notably, the EF-loops are highly conserved in ZTL and FKF1 proteins, but divergent between the two family members. Thus, it is likely that the EF-loop may be important in differentiating signaling members within this family. Further studies of the EF-loop and EF-loop variants can clarify how these underlying characterized structural motifs dictate LOV signal transduction and regulation of protein-protein interactions.

Notably, the EF-loop is unique to members of the ZTL family, and fungal circadian clock photoreceptors VVD and White-Collar 1. Computational studies of both families now highlight coupling between N-terminal signaling regions and EF-loop dynamics, however with reversed topology. Specifically in ZTL, light drives ordering of the N/C-termini and resultant restriction on EF-loop dynamics. In contrast, although VVD was found to follow the same two steps, they occur in the reverse order.[19] The variation in the order in signaling events is consistent with the unique signaling mechanism of ZTL, where light represses the activity of the C-terminal E3-ligase activity in contrast to light-regulated increases in activity of most LOV proteins. Overall, our combined MSM/ML platform allows unique insight into how the LOV signaling mechanism can follow conserved pathways but modulate both the direction and magnitude of signaling responses depending on residue substitutions at key allosteric sites.

### Allosteric variants and alteration to dynamics-based signaling

Structural approaches to identify allosteric mechanisms often rely on identifying allosteric variants that can trap the signaling protein in various presumed signaling states. In ZTL, proposed mechanisms were dependent on an allosteric variant (V48I:G80R) trapped a dark-state like conformation and an allosteric variant (G46S:G80R) trapped in a putative light-state like conformation. Although these structural approaches provide keen insights into both proximal and global conformational changes mediating signal transduction, their dependence on static-structures masks dynamics-based impacts on signal transduction, and may reflect artifacts due to an altered signaling mechanism caused by the mutations. In ZTL, this led to an unexplained aspect of the G46S variant, that if G46S:G80R already adopts the light-state parallel dimer, how does light enhance protein-protein interactions with its target GIGANTEA?

Here, a combined MD/MSM/ML platform provides direct insight into how allosteric variants alter protein dynamics and perturb a dynamics-based allosteric mechanism. We demonstrate how two variants have different effects on signal transduction. In the case of V48I, perturbation of the allosteric mechanism results directly from impeding Gln154 conformational dynamics necessary for sampling N/C-termini conformational states which are necessary for downstream allosteric responses. In contrast, G46S variants directly alter the entirety of the allosteric mechanism leading to sampling of only the light-state conformational landscape. In this altered mechanism, the steric bulk of Ser46 alters the interaction between Gln154 and Phe156, which perturbs both C- and N-termini dynamics. The altered interactions permit sampling of a unique (extended) Gln154 conformation that enhances sampling of the light-state landscape and leads to a light-dependent enhancement in GI interactions. In both cases, the variants selected are modest mutations widely tolerated in the LOV protein family, but uniquely evolutionarily selected for in ZTL. The ability of two residues in close proximity to direct unique alterations in signal transduction highlights the importance of combined structural and computational approaches in designing or studying new members of domain families.

## Materials and methods

### Molecular dynamics simulations

The initial structures of the dark and light states for the ZTL dimer complex were taken from the Protein Data Bank (PDB): the wild type (WT) dark state in anti-parallel conformation (PDB ID: 5SVG), the light and dark anti-parallel conformations of V48I:G80R (PDB ID: 5SVV and 5SVW, respectively), and the G46S:G80R mutant in light parallel conformations (PDB ID: 6WLP). The light state WT anti-parallel and transient dark anti-parallel structures were constructed based on the light V48I:G80R mutant crystal structure. The WT Parallel structures were constructed based on the G46S:G80R mutant crystal structure. From the native dark and light crystal structures, two transient structures were generated. The transient dark structure was generated by breaking the covalent bond between the Cys82 and the FMN, in the native light structures of ZTL. The transient light structure was generated by forming the Cys82-FMN bond and protonating FMN N5 in the dark state structures of ZTL. The structures subjected to simulations in this study are listed in Table 1.

To maintain the same number of residues for all structures, the termini of the monomers in native dark and light states were modeled to be the same length. The total number of residues per monomer is 130. All structures subjected to simulations in this study contain FMN as cofactor. The parameters used for the cofactor were taken from a previous study.[22] Hydrogen atoms were added to the crystal structures. The protonation states for the histidines were assigned using the ProteinPrepare tool of PlayMolecule.[52] The structures were then solvated using TIP3P water molecules and neutralized by adding chloride anions and sodium cations. The structures were minimized first with the steep descent method for 200 steps and the adopted basis Newton-Raphson minimization for 1000 steps afterwards. An initial 24 ps molecular dynamics was carried to increase the temperature from 0K to 300K.

For each structure, three 10 ns isothermal-isobaric ensemble (NPT) equilibration dynamics started with random velocities were carried out. The final coordinates and velocities were used to start a production phase of 600 ns simulations of constant-volume constant-temperature (NVT) ensemble. In the production simulations, the first 100 ns were considered as equilibration and therefore excluded from the analysis. A total of 15 *μ*s of MD trajectories have been generated. The simulations were carried using OpenMM 7.3 on GPU CUDA accelerated.[53] The NPT simulations were performed using a MonteCarloBarostat implemented in OpenMM 7.3. The NVT simulations were performed using the Langevin integrator. For the integrator, a friction coefficient of 1 ps^-1^ was implemented.[53] In all simulations, the SHAKE constraint for hydrogen covalent bonds was applied.[54] A step size of 2 fs was used. A triclinic box was used in the simulations. Period boundary conditions were applied. The particle mesh Ewald method was used to calculate the electrostatic interactions.[55]

### Trajectories analysis

The convergence and the stability of the trajectories were assessed through the analysis of the Root Mean Squared Deviation (RMSD) of the Cartesian coordinates of all atoms between each frame from the simulation superimposed onto the first frame of the simulation as the reference structure. The RMSD for a given simulation is defined as:

$$RMSD=\sqrt{\frac{\sum_{i=1}^{N}\left(r_{i}^{0}-Ur_{i}\right)}{N}},$$ (1)

where N is the number of atoms, *r*_*i*_ and *r*_*i*_^0^ are the Cartesian coordinate vectors for atom *i* in a simulated conformation and reference structure, respectively. *U* is the best-fit alignment transformation matrix between a given structure and its reference structure.

The Root Mean Squared Fluctuation (RMSF) of atom *i* is calculated as its averaged fluctuation.

$${RMSF}_{i}=\sqrt{\frac{1}{T}\sum_{j=1}^{T}\left(r_{i}^{j}-\bar{r_{i}}\right)},$$ (2)

where *T* is the number of total frames, $r_{i}^{j}$ is the coordinate of atom *i* in frame *j*, and $\bar{r_{i}}$ is the average position of atom *i* in the given trajectory. The RMSD and RMSF analyses, together with the distance analyses presented in this study, were performed using the MDtraj analysis package implemented in python.[56] The hydrogen bonds analysis presented in this study was carried out using a python script to detect hydrogen bonds according to the Baker and Hubbard criteria.[57] The visualization of the protein structures in the different analysis was done using 3D Protein Imaging[58] and VMD 1.9.3.[59]

### Time-structure independent component analysis

Time-structure independent component analysis (tICA) was employed as the dimensionality reduction method to analyze the simulations. The tICA method maximizes the auto-correlation function, which results in finding the slowest degrees of freedom and therefore in preserving the kinetic information present in the trajectories.[60–63] Given a time-series of molecular coordinates provided by the MD trajectories *x*(*t*) = ^*t*^(*x*_1_(*t*),…,*x*_*n*_(*t*)), tICA aims to reduce the dimensionality of the trajectories and to identify hidden key structural changes by decomposing the generalized eigenvalue problem $\bar{C}F$ = *CFK*. Where *K* = *diag*(*k*_1_,…,*k*_*n*_) and *F* = (*f*_1_,…,*f*_*n*_) are the eigenvalue and eigenvector matrices, respectively. *C* and $\bar{C}$ are the covariance matrix and the time-lagged covariance matrix of the coordinate vector, respectively.


$$C=\langle {\left(x(t)-\langle x(t)\rangle \right)}^{t}(x(t)-\langle x(t)\rangle )\rangle$$ (3)

$$\bar{C}=\langle {\left(x(t)-\langle x(t)\rangle \right)}^{t}(x(t+t_{0})-\langle x(t)\rangle )\rangle$$ (4)

In order to obtain a symmetric time-lagged covariance matrix, $\frac{1}{2}(\bar{C}+{}^{t}\bar{C})$ is calculated. The latter step assumes the time reversibility of the process, which is satisfied in MD trajectories. Lastly, the projected vectors of the MD are:

$$a(t)={}^{t}(a_{1}(t),\ldots ,a_{n}(t))={}^{t}Fx(t)$$ (5)

The tICA method has been successfully applied for the kinetic analysis of MD simulations.[60–64] In this study, the first two components from tICA were selected for visualization, interpretation and further analysis. The identification of the tICA component was based on all the trajectories considered in this study. In computing the tICA components, the trajectories were first concatenated and 100 ps was used as lag time for maximal inclusion of atomistic correlated motions (S20 Fig). The featurization of the protein using all the C*α* pairwise distances and dimensionality reduction were performed using the MSMBuilder package.[65]

### Markov state model

Markov state models (MSMs)[29–32,66–68] have become increasingly useful network models with the continuously developing open source software infrastructure[65,69–71] for describing the transitions among functional states during allosteric events.[72–74] Combined with MD simulations, MSM approaches can provide connectivity maps of states on the free energy landscape, estimate the effect of allosteric perturbations in the conformational equilibrium, and rigorously describe kinetics of allosteric transitions. Recent advances in the field have highlighted how MSM tools can help to recognize structural and dynamic patterns of conformational ensembles, identify functional allosteric states hidden in the conformational ensembles and reconstruct allosteric mechanisms.[74] Markov models have been employed for understanding the reaction mechanisms, thermodynamics and free-energy landscape population shifts, the hierarchy of timescales and the structural basis of allosteric events.[19,29–34]

MSM is used to track the conditional transition probabilities among non-overlapping states. The collection of the transition probabilities among *n* states is the transition matrix $T_{ij}=\frac{c_{ij}}{\sum_{k}c_{ik}}$, where *c*_*ij*_ is the count of the number of times the trajectories transition from a state *i* to a state *j* within a certain time interval Δt, called lag time *τ*.

To build the MSM, the trajectories were clustered based on geometrical similarity into 150 clusters (S8 Fig) using MiniBatchKmeans implemented in MSMbuilder. The clustered trajectories were then fed to MSMBuilder 3.8.0 to construct kinetically relevant Markov states.

Using the transition matrix, the probability to find the system in any state after certain time *τ* can be calculated using the equation *p*(*t*+*τ*) = *p*(*t*)*T*(*τ*). The first two components of tICA were used as collective variables. MSMBuilder python package was used to build the MSM.[65] The default hyper-parameters provided by MSMBuilder were used for the analysis. The ergodic cutoff was turned on and the Maximum Likelihood method was used to achieve the reversibility of the transition matrix. An appropriate lag time for transitions among microstates was estimated based on the convergence of the implied relaxation timescale. A relaxation time scale can be interpreted as a time length needed for the system to reach a particular steady state.[75] The implied relaxation timescales obtained at various lag times show the convergence roughly for lag times longer than 30 ns (S8 Fig), which is chosen to build the final MSM.

A total of 11 macrostates were created using the Perron-cluster cluster analysis (PCCA) implemented in the MSMbuilder package.[65] This method is based on the assumption that states belonging to the same free energy basin will interconvert easily, providing a higher transition probability for these states. Therefore, the separation of the states can be extracted via spectral decomposition of the transition probability matrix.[76]

### Machine learning

The supervised machine learning method one-vs-one (OvO) random forest[77] implemented in the Scikit-learn python package[78] was used in this study. OvO Random Forest uses parameters Φ = (*j*,*t*) composed of the data features *j* and a threshold *t* to divide the data in two parts based on the threshold.

$$Q_{left}(\theta )=(x,y)|x_{j}\leq t,$$ (6)

$$Q_{right}(\theta )=(x,y)|x_{j}\geq t,$$ (7)

with *x* being the training data and *y* being the training label. The protein features utilized for this analysis were all the C*α* pairwise distances. The Gini impurity criterion was used to assess the quality of the model. The Gini impurity score represents the likelihood of an incorrect classification of a new random variable according to the existing distributions of the labels k:

$$G=\Sigma_{k}p_{k}(1-p_{k})$$ (8)

Decision tree methods suffer from possible bias towards certain set of features given a certain data batch.[79] The random forest approach overcomes this problem and ensures more statistical solidity by taking a collection of decision trees, which are composed of different data batches, and uses a majority voting system amongst the classification output of all the single trees.[80]

OvO random forest model is trained not on all the classes but for each pair of classes.[81] This provides the possibility to extract not only the features that are responsible for the distinguishability of the different classes (metastable states) and their importance, but also to compare each pair of metastable states as well. The number of estimators for an OvO Random Forest model is:

$$n_{estimators}=\frac{n_{classes}\times (n_{classes}-1)}{2}$$ (9)

A total of 55 estimators were built. The validation of the Random Forest hyper parameters was performed using the GridSearch method implemented in Scikit-learn. The values which provided the best accuracy for our model are 10 trees of 4 layers depth.

### Pearson correlation analysis

The Pearson correlation (PC) is a measure of linear correlation between two variables.[82] The first step in building the PC is calculating the covariance between two variables:

$$\sigma (x,y)=\frac{1}{n-1}\sum_{i=1}^{n}\left(x_{i}-\bar{x})(y_{i}-\bar{y}\right)$$ (10)

The covariance is then divided by the square root of the product of the variance of each variable.


$$\rho (x,y)=\frac{\sigma (x,y)}{\sqrt{Var(x)Var(y)}}$$ (11)

PC is dimensionless and can assume values in the range of [–1, +1], where the two extremes stand for total anti-correlation and total correlation, respectively.

## Supporting information

S1 Fig Root mean squared deviation analysis of MD simulations. a) Dark state structures; b) Light state structures. The replicas of the same structure are plotted with the same color.
(TIF)

S2 Fig Root mean squared fluctuation analysis of MD simulations. a) Dark state structures; b) Light state structures; c) RMSF illustration in protein structure. Cyan color represents low RMSF values, Red represents high RMSF values.
(TIF)

S3 Fig RMSD analysis for each structure individually. (TIF)

S4 Fig Visualization of chi1, chi2, and chi3 dihedral angles of the Gln154 residue. (TIF)

S5 Fig Hydrogen bond between residue Gln154 and Asn123 in: a) Dark states; b) Light states.
(TIF)

S6 Fig Mutations performed in the FMN binding site. a) Overlap of V48I mutation (orange) with WT (cyan). b) Overlap of G46S mutation (orange) with WT (cyan).
(TIF)

S7 Fig 2D projections made with tICA dimensionality reduction with different sets of Cα distances. a) Distances between Cα within monomer 1. B) Distances between Cα within monomer 2. c) Distances between Cα of monomer 1 and Cα of monomer 2.
(TIF)

S8 Fig Markov state model (MSM) and Perron-Cluster Cluster Analysis (PCCA) of ZTL dimer simulations. a) Estimated relaxation timescale. This is based on the transition probabilities among different microstates using different lag times. b) Distribution of 11 macrostates on a 2D tICA projection. The Potential Free energy of each macrostate was calculated based on the population.
(TIF)

S9 Fig Correlation between macrostates and ZTL functional states. a) Distribution of different functional states in each macrostate identified in the MSM; b) Using the states occupation the tICA subspace has been divided in four different regions: native dark region (blue), transient region (orange), native light region (dark green), and transient parallel region (purple).
(TIF)

S10 Fig Structural representation and density distribution of the selected features from the OvO random forest classification model. a) Top across-dimer feature (Cα distances between monomer A Ala45 and monomer B Leu33); b) Density distribution of the top across-dimer feature; c) Top within-monomer feature (monomer A Ala36 and Asn56); d) Density distribution of the top within-monomer feature; e) The least important feature (monomer A Leu78 and Gly121); f) Density distribution of the least important feature.
(TIF)

S11 Fig OvO random forest cumulative feature importance. 357 out of 33670 features account for 90% of distinguishability between macrostates.
(TIF)

S12 Fig Key structural factors associated with states changes in ZTL dimer. a) Undocking of the N-termini from macrostate 2 to 3 (top feature as monomer B residue Pro28 to Arg66); b) Reduction of the EF-loop flexibility from macrostate 3 to 9 (top feature as monomer B residue His89 to Pro123); c) Packing of N-termini from macrostate 9 to 4 (top feature as monomer B residue Gly30 to Lys102). For each step, the blue distributions represent the starting state and the orange distributions represent the end state.
(TIF)

S13 Fig Within-monomer and cross-dimer features importance along the allosteric path. (TIF)

S14 Fig OvO random forest model to differentiate the dark native WT antiparallel state and the V48I:G80R mutant. a) Structural representation of the most important cross-monomer distance (Monomer A Leu32 and monomer B Ile102); b) Density distribution of the most important cross-monomer distance; c) Structural representation of the most important within-monomer feature (Monomer A Val92 and Phe117); d) Density distribution of the most important within-monomer feature.
(TIF)

S15 Fig OvO random forest model to differentiate the Light native WT parallel state and the G46S:G80R mutant (Monomer A Lys102 and monomer B Thr35): a) Structural representation; b) Density distribution.
(TIF)

S16 Fig Structural differences between the dark native WT anti-parallel and the V48I:G80R mutant. Overlap between representative conformations of State 2 (Native Dark WT anti-parallel) and State 7 (V48I:G80R mutant). Color map indicates structure similarity based on RMSD. Blue represents similarity and red represents dissimilarity. The biggest difference is the unfolding and undocking of the N-termini in the mutant structure.
(TIF)

S17 Fig Structural differences between the Native Light WT parallel and the G46S:G80R mutant. Overlap between representative conformations of State 4 (Native Light WT parallel) and State 6 (G46S:G80R mutant). Color map indicates structure similarity based on RMSD. Blue represents similarity and red represents dissimilarity.
(TIF)

S18 Fig Light dependent Iβ strand pull investigation of Q154L mutants. Distribution of Phe156 CA and FMN(O4) distance for ZTL structures.
(TIF)

S19 Fig Distance analysis between Phe66 and FMN for ZTL Q154L variants in different light conditions. The structures were the flavin is modeled in the light state show smaller distances between Phe66 and FMN, showing a possible attraction between this residue and the photoreceptor depending on the light condition.
(TIF)

S20 Fig Projection of the first 2 tICA components at different tICA lag-times. (TIF)

S1 Table Representative sets of dihedral angles used for the dihedral based Gln154 conformation classification analysis. (XLSX)

S2 Table Phe156 pull, Gln154 rotation Pearson Correlation analysis. (XLSX)

S3 Table Transition probability matrix between States identified in the MSM. (XLSX)

S1 Text Protein Stability Analysis. (DOCX)
