# Supplementary figures and images for: Dimeric allostery mechanism of the plant circadian clock photoreceptor ZEITLUPE
Source: PLoS Comput Biol. 2021 Jul 26;17(7):e1009168. doi: 10.1371/journal.pcbi.1009168 (PMC8341706; doi:10.1371/journal.pcbi.1009168)

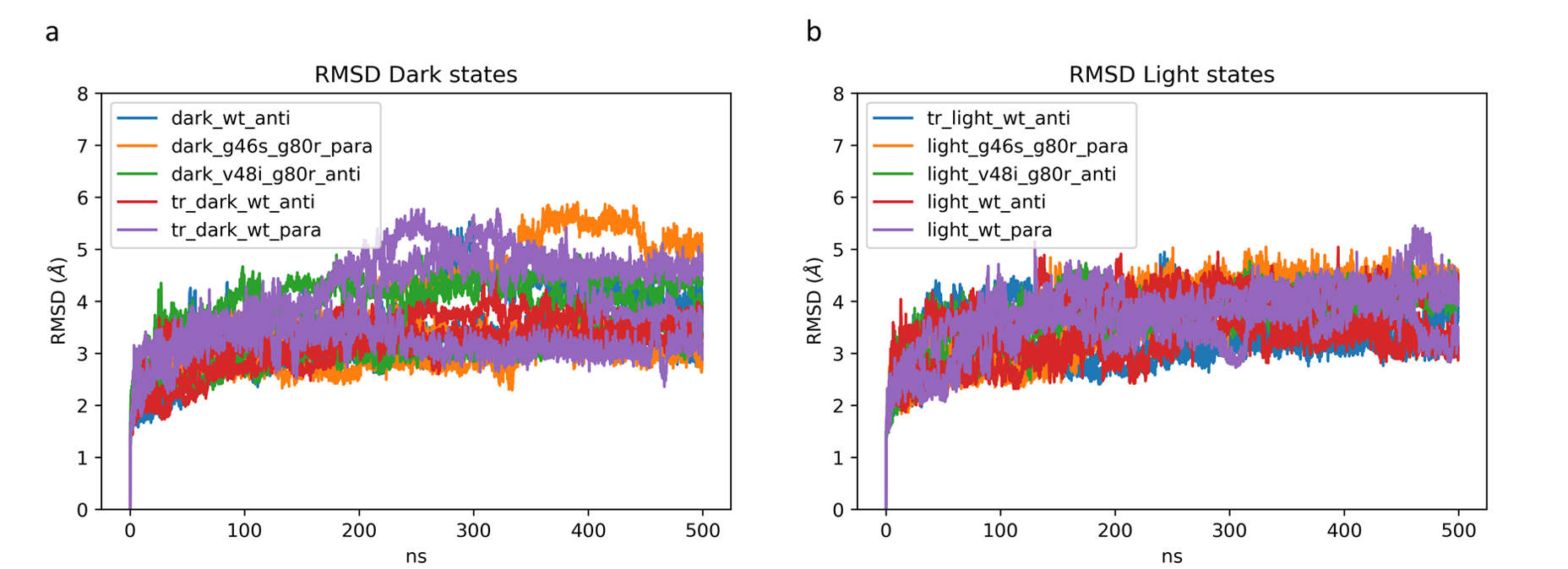

Supplement: S1 Fig — a) Dark state structures; b) Light state structures. The replicas of the same structure are plotted with the same color. (TIF) [file pcbi.1009168.s001.tif]

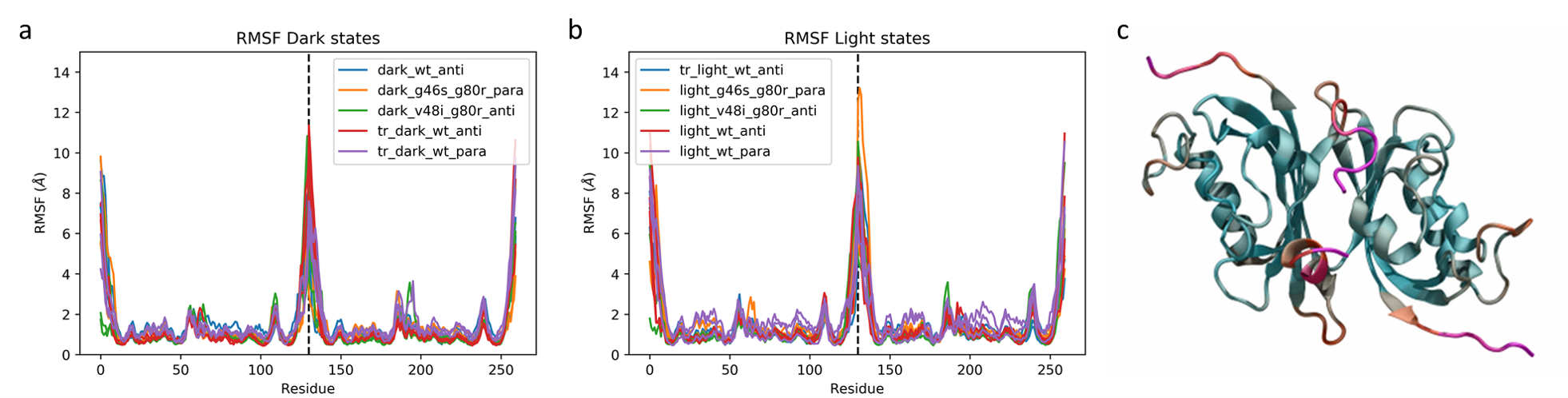

Supplement: S2 Fig — a) Dark state structures; b) Light state structures; c) RMSF illustration in protein structure. Cyan color represents low RMSF values, Red represents high RMSF values. (TIF) [file pcbi.1009168.s002.tif]

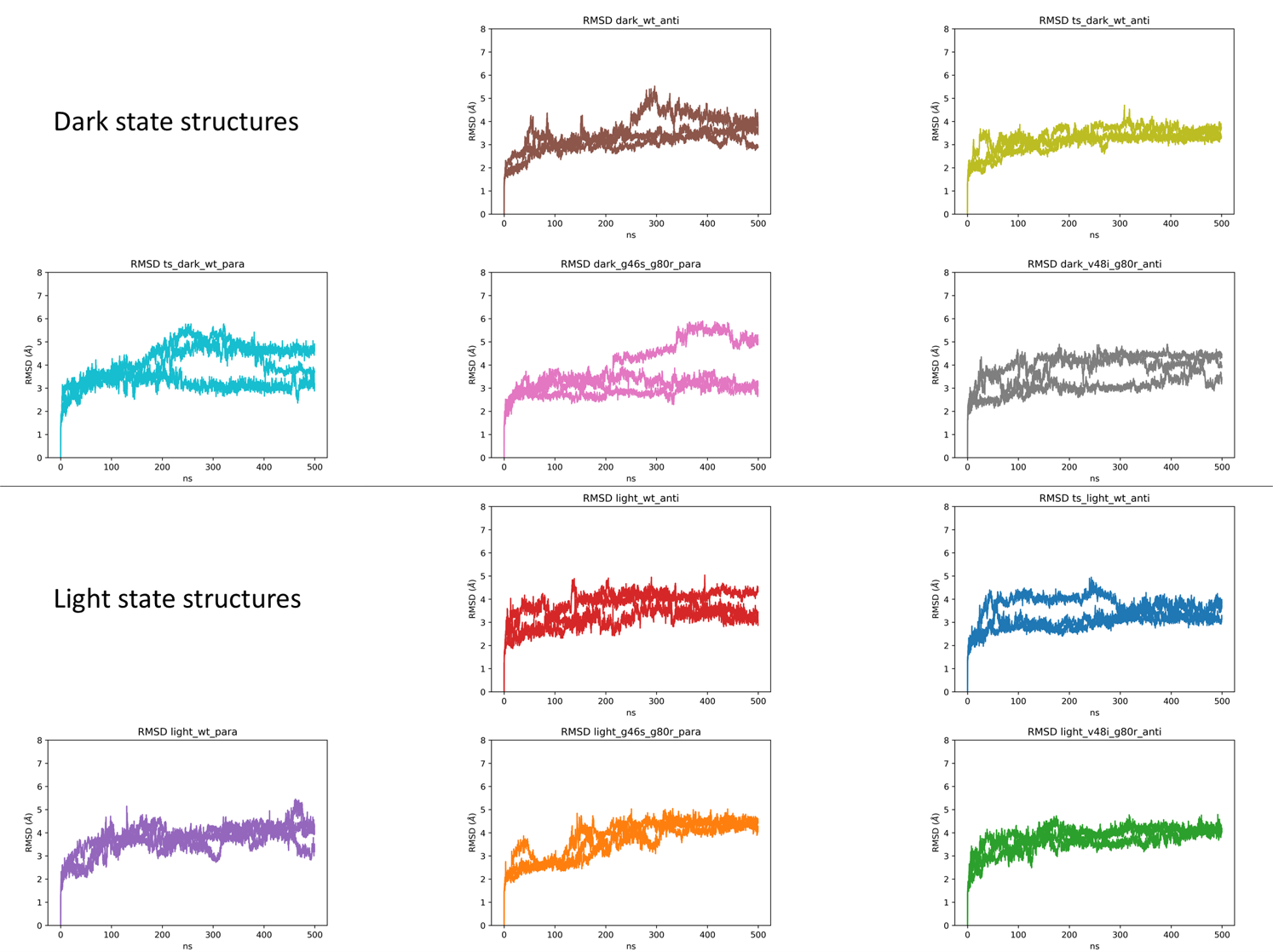

Supplement: S3 Fig — (TIF) [file pcbi.1009168.s003.tif]

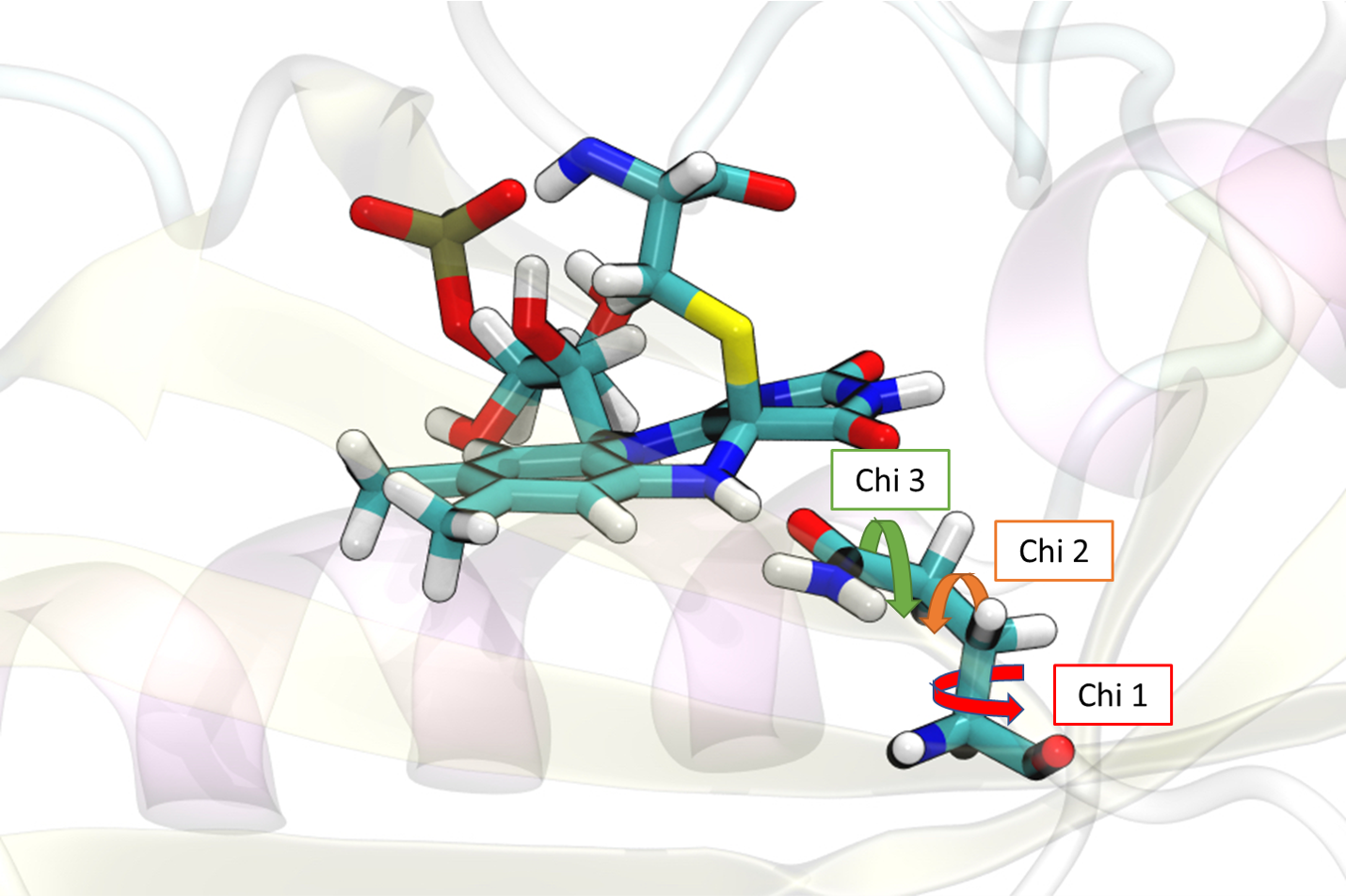

Supplement: S4 Fig — (TIF) [file pcbi.1009168.s004.tif]

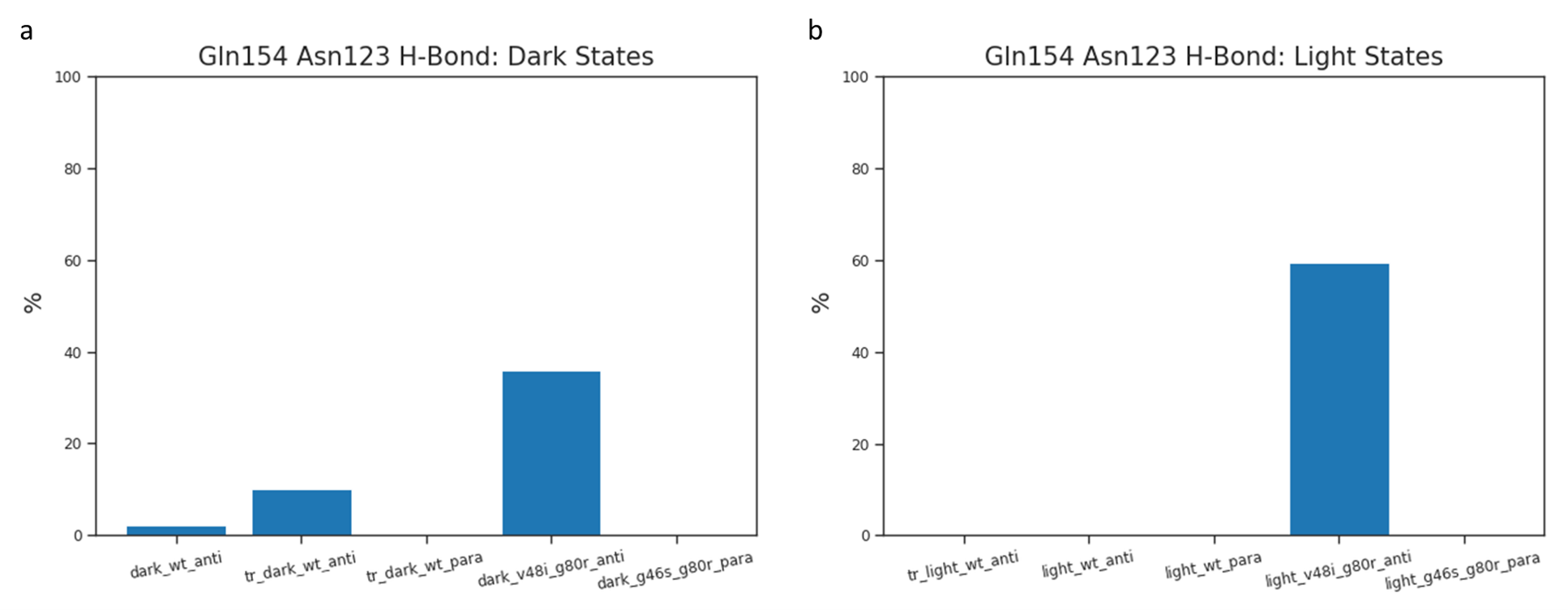

Supplement: S5 Fig — Hydrogen bond between residue Gln154 and Asn123 in: a) Dark states; b) Light states. (TIF) [file pcbi.1009168.s005.tif]

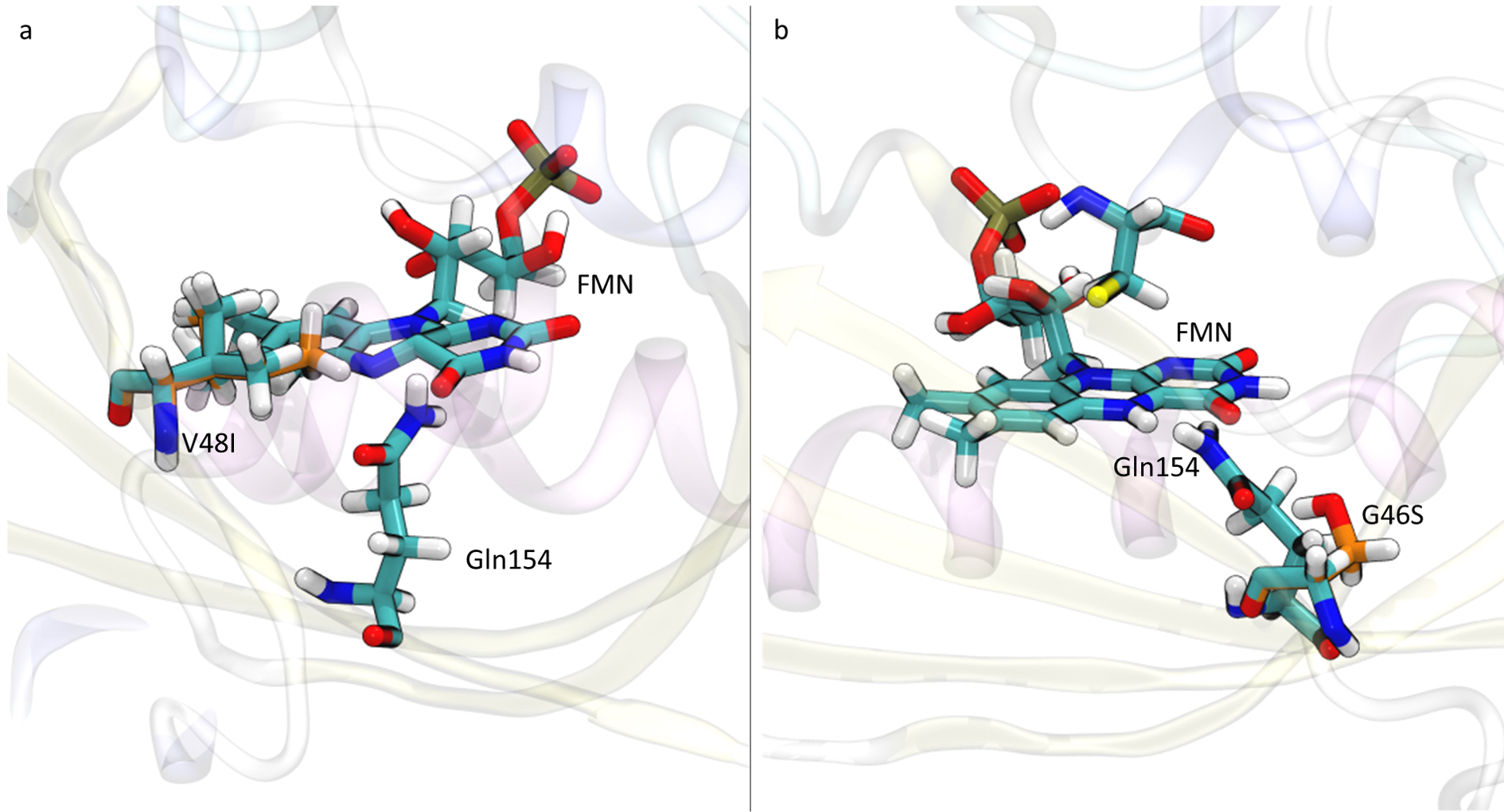

Supplement: S6 Fig — a) Overlap of V48I mutation (orange) with WT (cyan). b) Overlap of G46S mutation (orange) with WT (cyan). (TIF) [file pcbi.1009168.s006.tif]

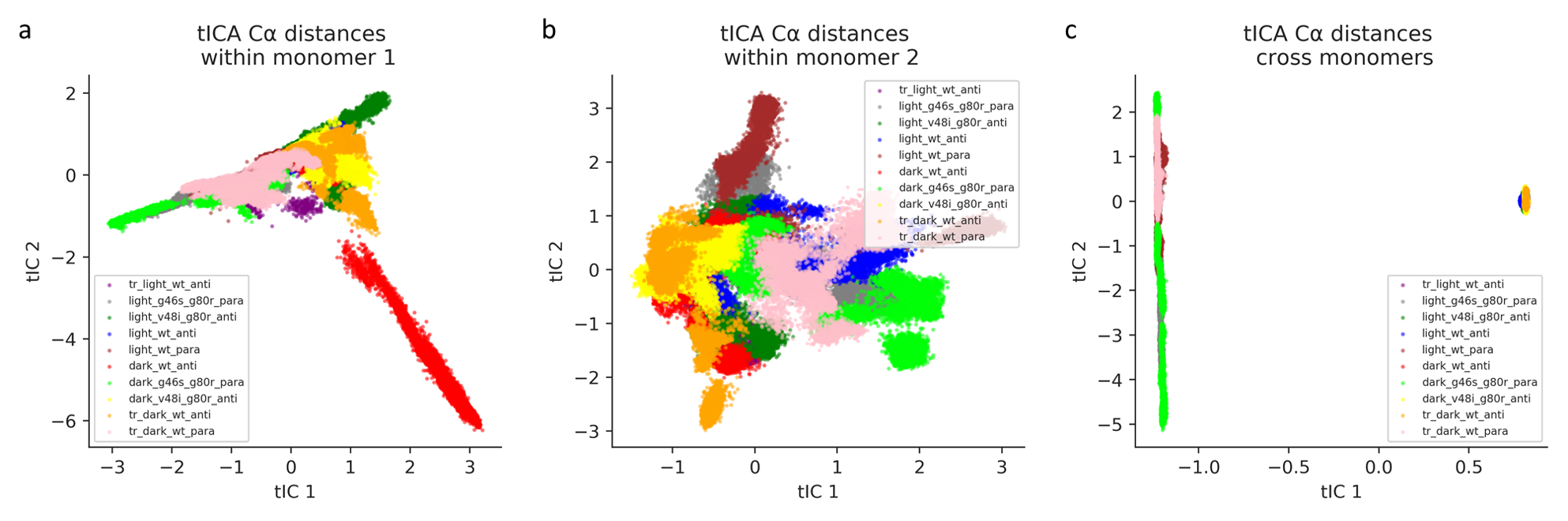

Supplement: S7 Fig — a) Distances between Cα within monomer 1. B) Distances between Cα within monomer 2. c) Distances between Cα of monomer 1 and Cα of monomer 2. (TIF) [file pcbi.1009168.s007.tif]

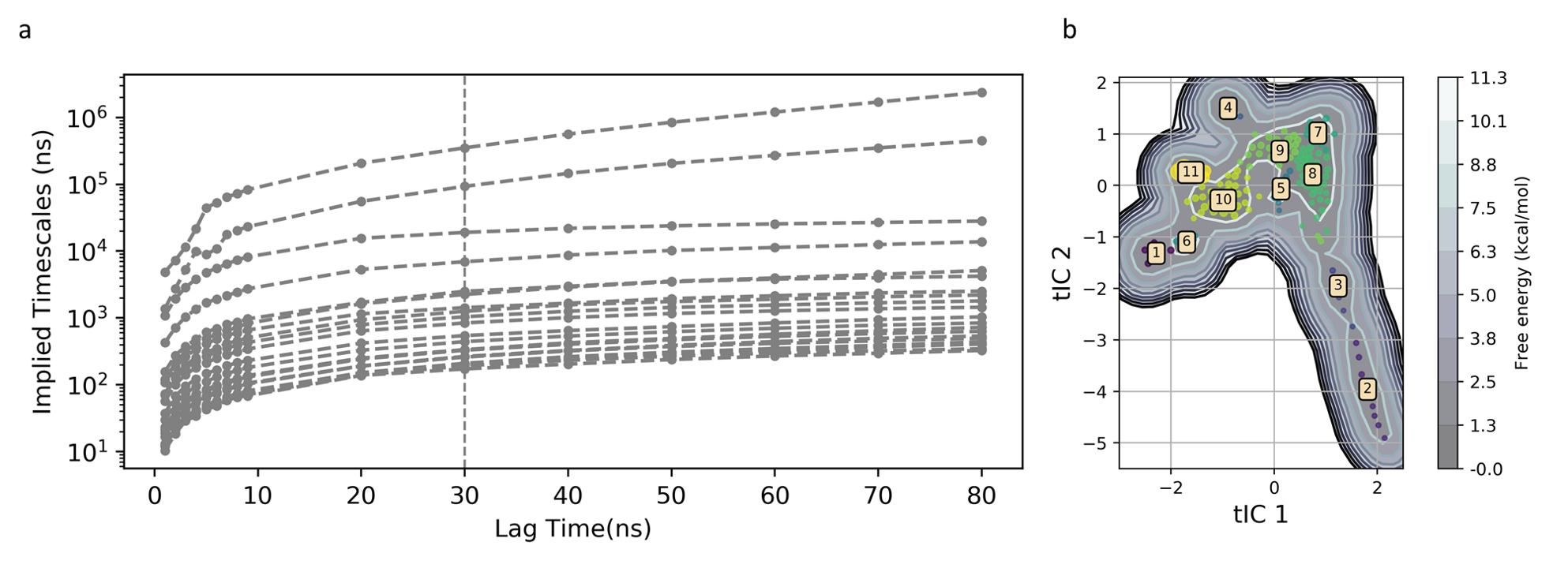

Supplement: S8 Fig — a) Estimated relaxation timescale. This is based on the transition probabilities among different microstates using different lag times. b) Distribution of 11 macrostates on a 2D tICA projection. The Potential Free energy of each macrostate was calculated based on the population. (TIF) [file pcbi.1009168.s008.tif]

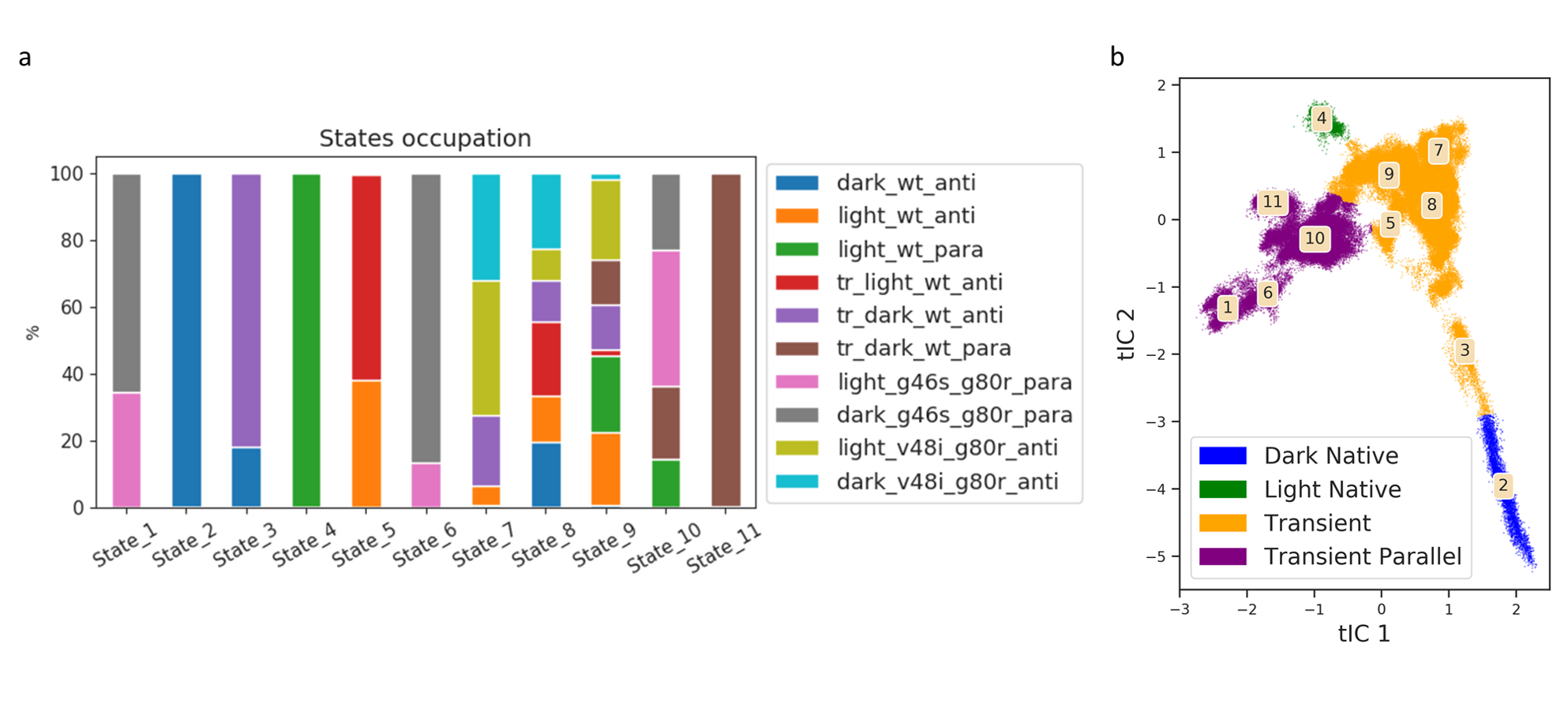

Supplement: S9 Fig — a) Distribution of different functional states in each macrostate identified in the MSM; b) Using the states occupation the tICA subspace has been divided in four different regions: native dark region (blue), transient region (orange), native light region (dark green), and transient parallel region (purple). (TIF) [file pcbi.1009168.s009.tif]

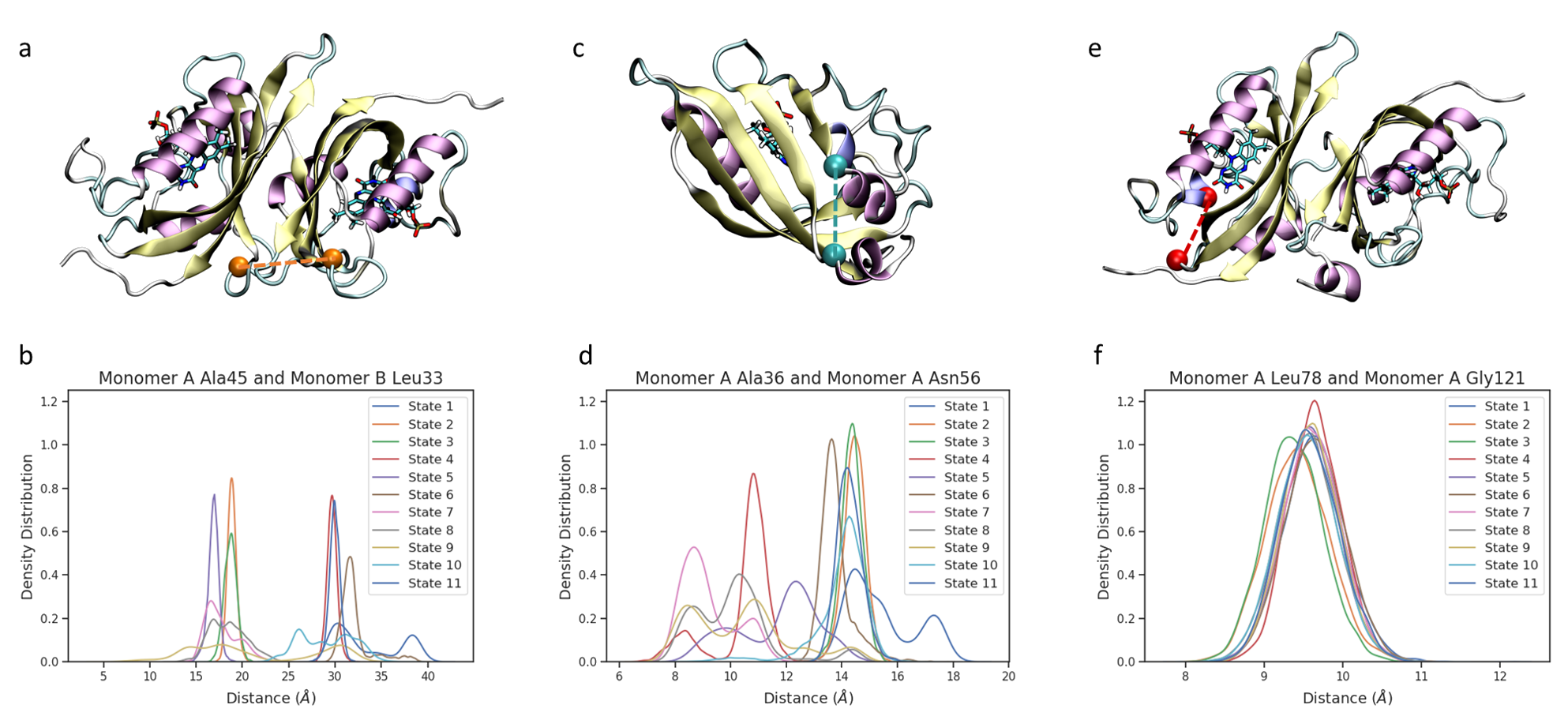

Supplement: S10 Fig — a) Top across-dimer feature (Cα distances between monomer A Ala45 and monomer B Leu33); b) Density distribution of the top across-dimer feature; c) Top within-monomer feature (monomer A Ala36 and Asn56); d) Density distribution of the top within-monomer feature; e) The least important feature (monomer A Leu78 and Gly121); f) Density distribution of the least important feature. (TIF) [file pcbi.1009168.s010.tif]

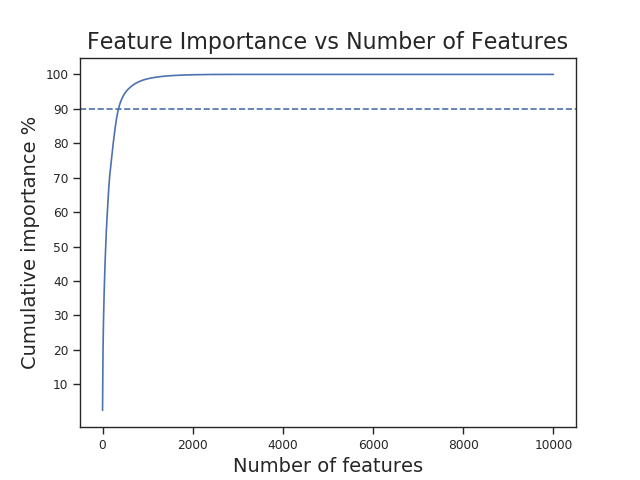

Supplement: S11 Fig — 357 out of 33670 features account for 90% of distinguishability between macrostates. (TIF) [file pcbi.1009168.s011.tif]

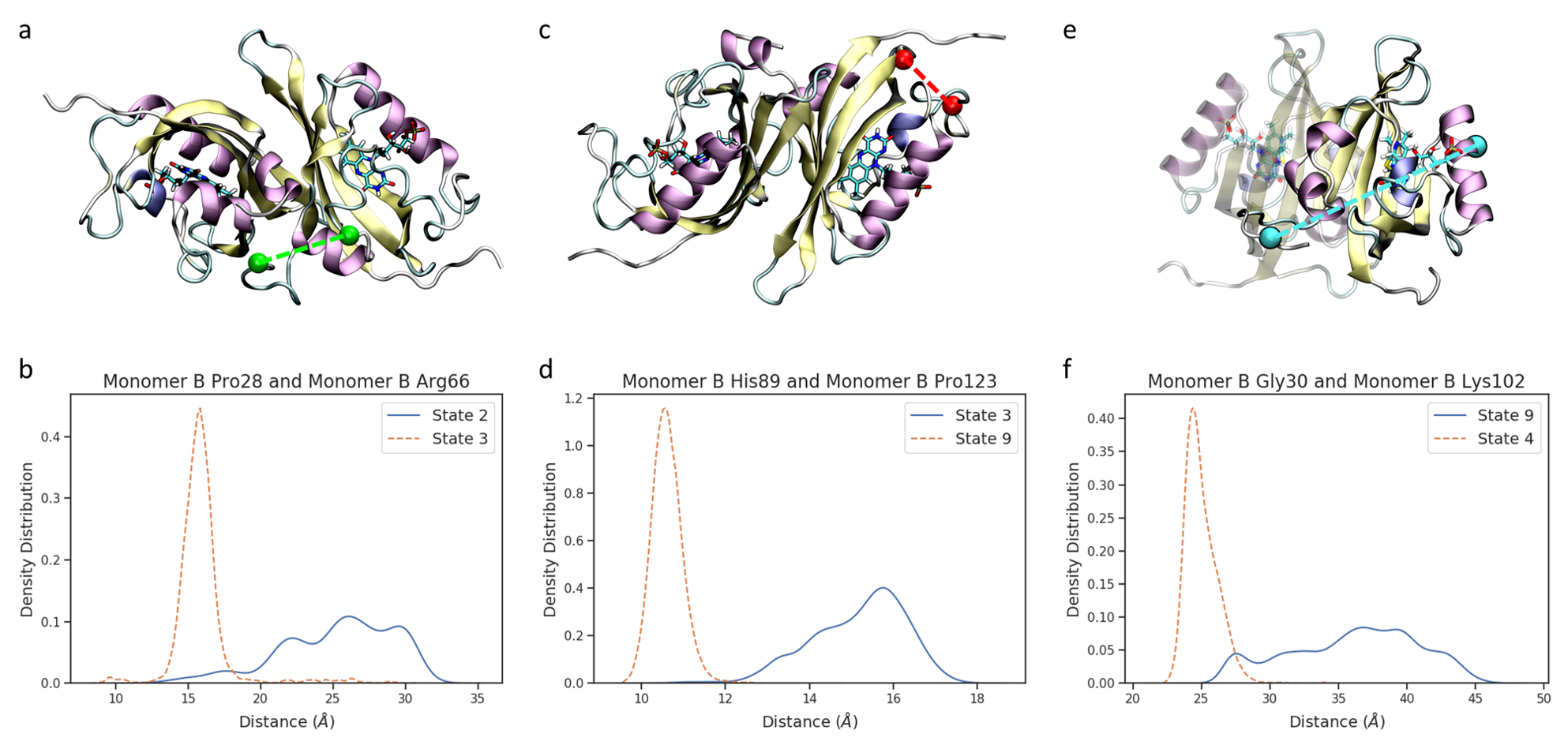

Supplement: S12 Fig — a) Undocking of the N-termini from macrostate 2 to 3 (top feature as monomer B residue Pro28 to Arg66); b) Reduction of the EF-loop flexibility from macrostate 3 to 9 (top feature as monomer B residue His89 to Pro123); c) Packing of N-termini from macrostate 9 to 4 (top feature as monomer B residue Gly30 to Lys102). For each step, the blue distributions represent the starting state and the orange distributions represent the end state. (TIF) [file pcbi.1009168.s012.tif]

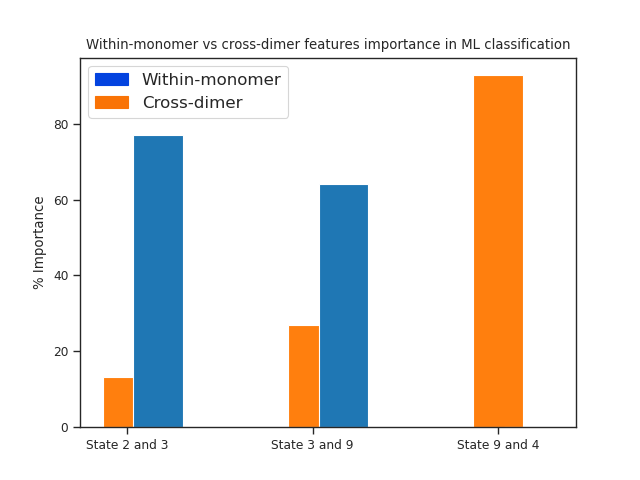

Supplement: S13 Fig — (TIF) [file pcbi.1009168.s013.tif]

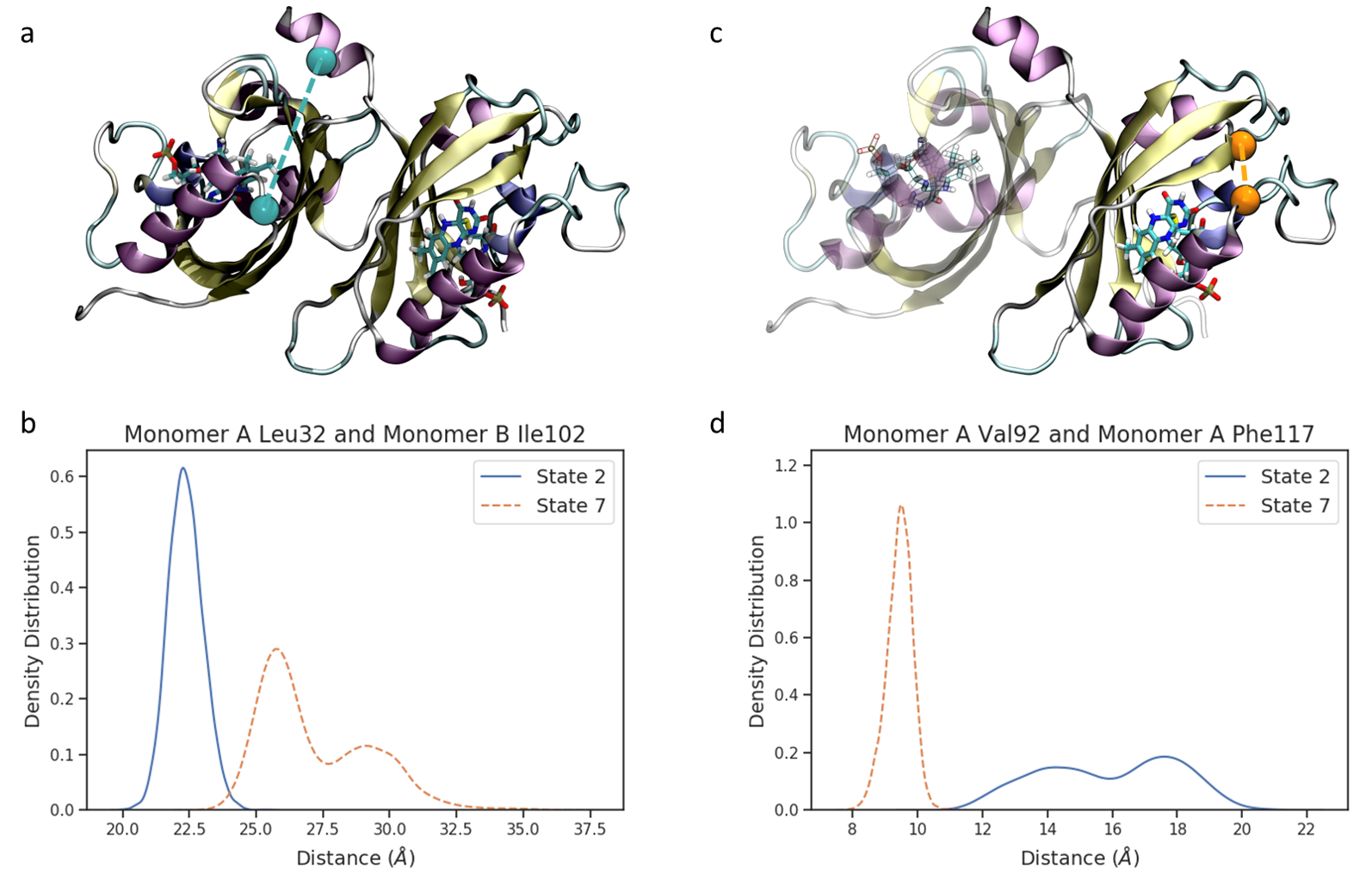

Supplement: S14 Fig — a) Structural representation of the most important cross-monomer distance (Monomer A Leu32 and monomer B Ile102); b) Density distribution of the most important cross-monomer distance; c) Structural representation of the most important within-monomer feature (Monomer A Val92 and Phe117); d) Density distribution of the most important within-monomer feature. (TIF) [file pcbi.1009168.s014.tif]

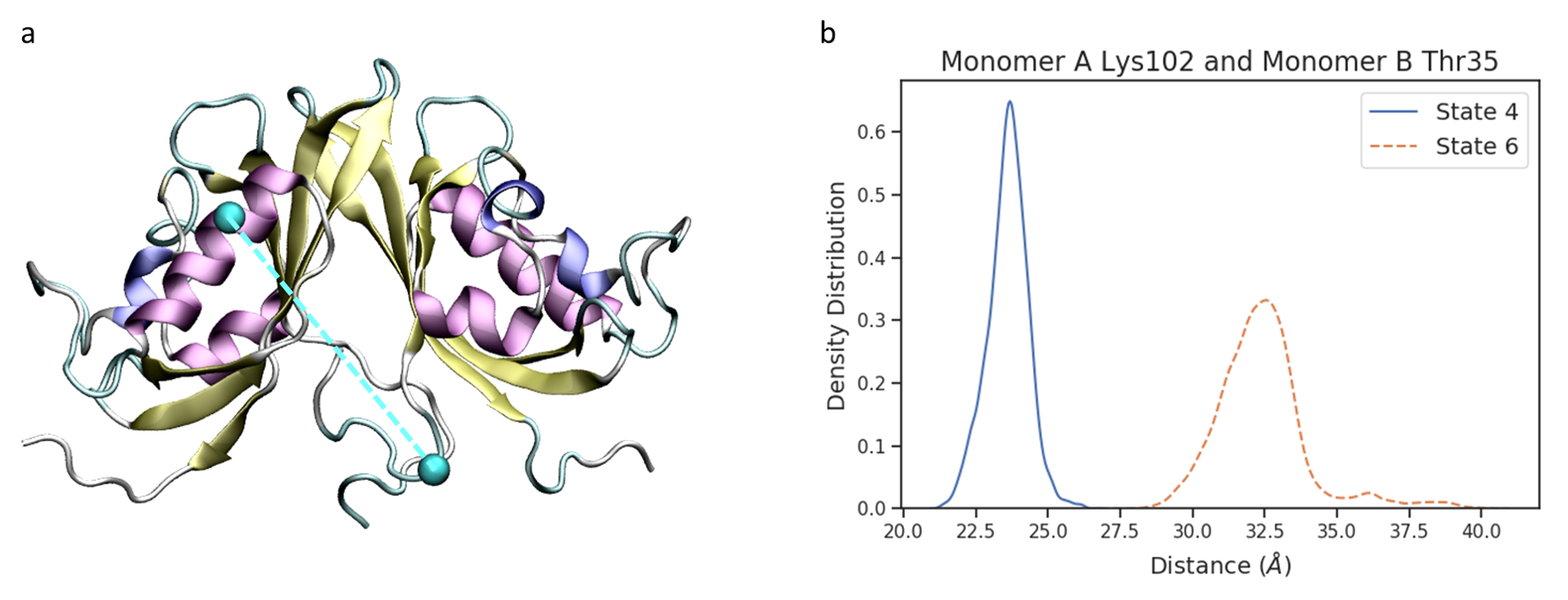

Supplement: S15 Fig — OvO random forest model to differentiate the Light native WT parallel state and the G46S:G80R mutant (Monomer A Lys102 and monomer B Thr35): a) Structural representation; b) Density distribution. (TIF) [file pcbi.1009168.s015.tif]

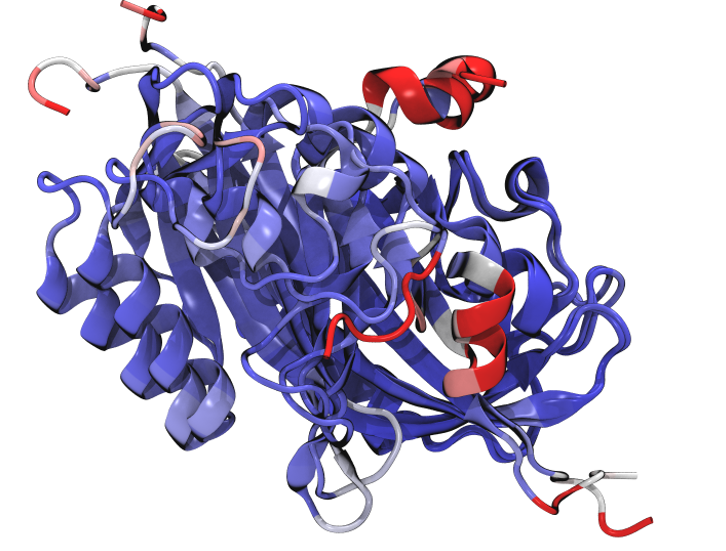

Supplement: S16 Fig — Overlap between representative conformations of State 2 (Native Dark WT anti-parallel) and State 7 (V48I:G80R mutant). Color map indicates structure similarity based on RMSD. Blue represents similarity and red represents dissimilarity. The biggest difference is the unfolding and undocking of the N-termini in the mutant structure. (TIF) [file pcbi.1009168.s016.tif]

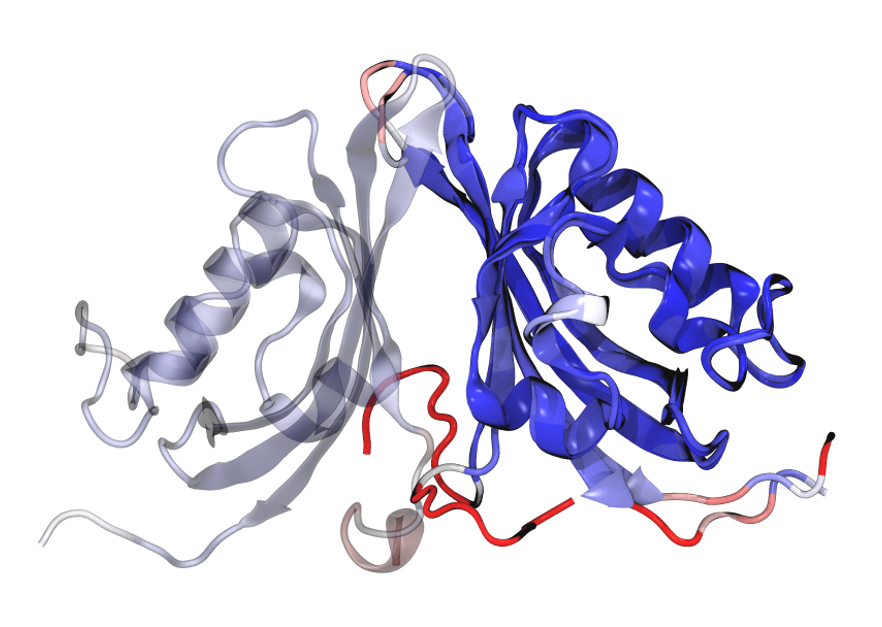

Supplement: S17 Fig — Overlap between representative conformations of State 4 (Native Light WT parallel) and State 6 (G46S:G80R mutant). Color map indicates structure similarity based on RMSD. Blue represents similarity and red represents dissimilarity. (TIF) [file pcbi.1009168.s017.tif]

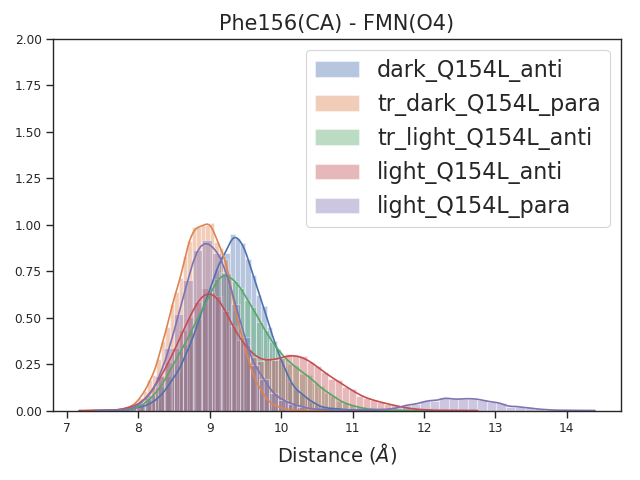

Supplement: S18 Fig — Distribution of Phe156 CA and FMN(O4) distance for ZTL structures. (TIF) [file pcbi.1009168.s018.tif]

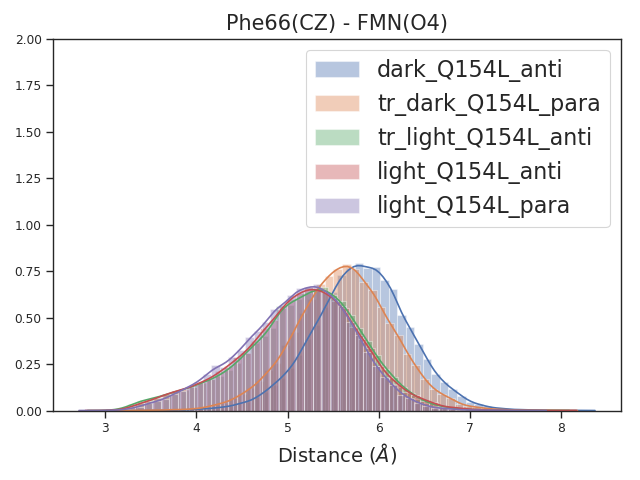

Supplement: S19 Fig — The structures were the flavin is modeled in the light state show smaller distances between Phe66 and FMN, showing a possible attraction between this residue and the photoreceptor depending on the light condition. (TIF) [file pcbi.1009168.s019.tif]

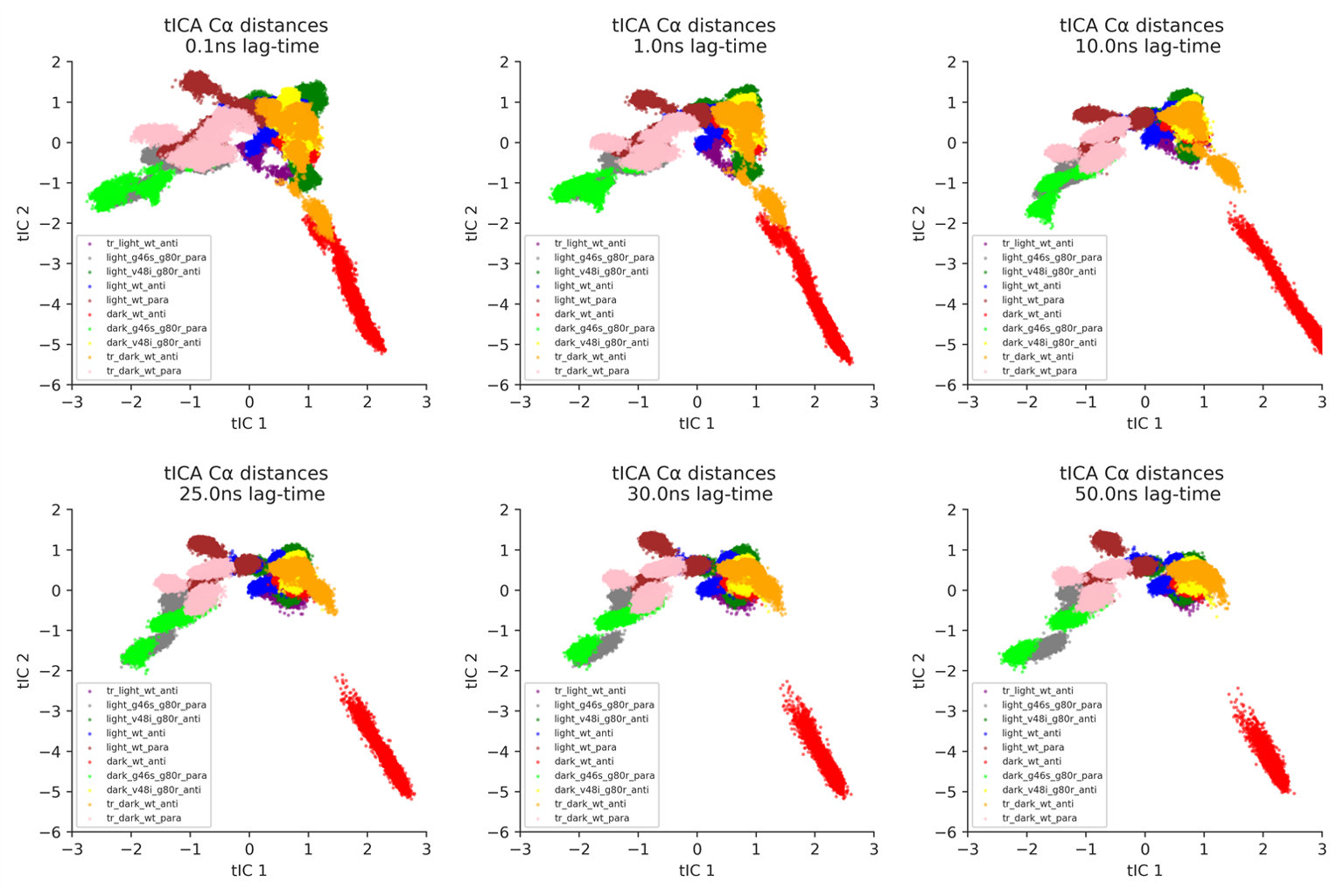

Supplement: S20 Fig — (TIF) [file pcbi.1009168.s020.tif]
