# Supplementary material for: Dimeric allostery mechanism of the plant circadian clock photoreceptor ZEITLUPE
Source: PLoS Comput Biol. 2021 Jul 26;17(7):e1009168. doi: 10.1371/journal.pcbi.1009168 (PMC8341706; doi:10.1371/journal.pcbi.1009168)
Supplement: S1 Text — (DOCX) [file pcbi.1009168.s024.docx]

## **Protein Stability Analysis**

MD simulations of WT and ZTL allosteric variants support dark-state anti-parallel, and light-state parallel conformations of the ZTL LOV dimer in WT proteins. The RMSD values, presented in S1 Fig, demonstrate overall stability of the structures during the MD simulations. In the dark state structures, where there is no covalent bond between the cofactor and the proximal Cys82 and the FMN N5 is deprotonated, the parallel structures show more fluctuation before converging (S1 Fig and purple and orange lines and S3 Fig). All the light state trajectories are well converged. This result indicates low conformational flexibility of the ZTL light state structures, as hypothesized by Pudasaini et al.[1]

The experimental characterization of the ZTL parallel dimer was obtained via G46S:G80R allosteric variant. Here, we assessed the stability of the recently identified parallel dimer interface in the WT. During the trajectories, the parallel oriented interface remains intact. In the light states, the parallel dimer interface remains stable during the entire course of the simulations, while in the dark state higher fluctuation of the RMSD values might indicate destabilization of the parallel interface, suggesting that the parallel conformation is consistent with ZTL light state.

The RMSF values, presented in S2 Fig show similar dynamic fingerprint of the secondary structure regardless the light condition and mutations. The structural regions with higher flexibility were found to be the N-termini, C-termini, and EF-loop (S2C Fig). This result indicates the involvement of these structural parts into functional dynamics and allostery of ZTL.

**References**

1. Pudasaini A, Shim JS, Song YH, Shi H, Kiba T, Somers DE, et al. Kinetics of the LOV domain of ZEITLUPE determine its circadian function in Arabidopsis. Elife. 2017;6:e21646.
